# MXene Sediment-Based Poly(vinyl alcohol)/Sodium Alginate Aerogel Evaporator with Vertically Aligned Channels for Highly Efficient Solar Steam Generation

**DOI:** 10.1007/s40820-024-01433-1

**Published:** 2024-06-17

**Authors:** Tian Wang, Meng Li, Hongxing Xu, Xiao Wang, Mingshu Jia, Xianguang Hou, Shuai Gao, Qingman Liu, Qihang Yang, Mingwei Tian, Lijun Qu, Zhenhua Song, Xiaohu Wu, Lili Wang, Xiansheng Zhang

**Affiliations:** 1 Shandong Key Laboratory of Medical and Health Textile Materials, College of Textiles and Clothing, State Key Laboratory of Bio-Fibers and Eco-Textiles, Research Center for Intelligent and Wearable Technology, Qingdao University, Qingdao, 266071 People’s Republic of China; 2 Department of Pharmacology, Qingdao University School of Pharmacy, Qingdao University, Qingdao, 266071 People’s Republic of China; 3 Shandong Institute of Advanced Technology, Jinan, 250100 People’s Republic of China; 4 College of Electromechanical Engineering, Qingdao University of Science and Technology, Qingdao, 266061 People’s Republic of China

**Keywords:** MXene sediments, Porous structure, Desalination, Self-floating

## Abstract

Solar-driven interfacial evaporation from seawater is considered an effective way to alleviate the emerging freshwater crisis because of its green and environmentally friendly characteristics. However, developing an evaporator with high efficiency, stability, and salt resistance remains a key challenge. MXene, with an internal photothermal conversion efficiency of 100%, has received tremendous research interest as a photothermal material. However, the process to prepare the MXene with monolayer is inefficient and generates a large amount of “waste” MXene sediments (MS). Here, MXene sediments is selected as the photothermal material, and a three-dimensional MXene sediments/poly(vinyl alcohol)/sodium alginate aerogel evaporator with vertically aligned pores by directional freezing method is innovatively designed. The vertical porous structure enables the evaporator to improve water transport, light capture, and high evaporation rate. Cotton swabs and polypropylene are used as the water channel and support, respectively, thus fabricating a self-floating evaporator. The evaporator exhibits an evaporation rate of 3.6 kg m^−2^ h^−1^ under one-sun illumination, and 18.37 kg m^−2^ of freshwater is collected in the condensation collection device after 7 h of outdoor sun irradiation. The evaporator also displays excellent oil and salt resistance. This research fully utilizes “waste” MS, enabling a self-floating evaporation device for freshwater collection.

**Supplementary Information:**

The online version contains supplementary material available at 10.1007/s40820-024-01433-1.

## Highlights

- MXene sediments is innovatively used as photothermal material for seawater desalination.
- Inspired by the natural wood transpiration process, a 3D MXene sediment-based aerogel with vertically aligned channels is innovatively prepared as solar evaporator.
- With unique structure and composition, excellent photothermal conversion efficiency, evaporation rate, salt resistance, and resistance to biological/oil/special environments are achieved in practical desalination.

**Supplementary Information:**

The online version contains supplementary material available at 10.1007/s40820-024-01433-1.

## Graphical abstract

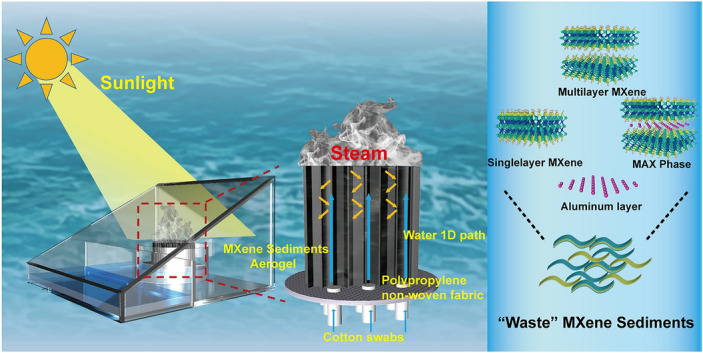

## Introduction

As the world’s population steadily increases, the demand for freshwater resources is also on the rise; however, only 0.3% of the world’s freshwater is available for direct human consumption [1]. Traditional desalination technologies such as low-temperature multi-effect distillation (LT-MED), multi-stage flash evaporator, reverse osmosis, and electrodialysis have disadvantages such as pipeline scaling, low efficiency, high energy consumption, and large footprint [2–4]. In recent years, solar-driven interfacial evaporation systems have gained significant research interest in the field of seawater desalination. These systems minimize heat loss by positioning solar energy on an evaporator surface made of photothermal materials [5–7]. This increases the efficiency of photothermal conversion and improves the evaporation rate of the desalination evaporator, resulting in low energy consumption [8], pollution-free, and convenient desalination technology. It is now one of the effective methods to solve the global of water scarcity [9].

When it comes to interface solar evaporation systems, researchers primarily focus on the selection of evaporator materials, water transmission channels, and evaporator structure design [10]. There has been a wide range of development in the materials used for interface solar evaporators, including nanofiber membranes [11], fabrics [12], sponges [13], hydrogels [14], and aerogels [15]. Aerogel-based materials is increasingly popular for use in solar steam applications due to their high porosity, which prevents the collapse of three-dimensional (3D) structures by trapping gas in the polymer network [16]. This property gives them high mechanical strength [17]. Additionally, their high specific surface area, low bulk density, and very low thermal conductivity make them ideal for use in evaporators in interfacial solar evaporation systems [18]. In these systems, the porous structure of aerogels, act as water transport channel, providing channels for efficient and continuous water evaporation. Therefore, the vertical porous structure porous structure of aerogels plays a crucial role evaporation process. The 3D water channel is directly connected to the water body, which makes it difficult to prevent energy loss during the transmission process. This results in a weakened thermal localization effect. To overcome this challenge, the team headed Zhu developed two dimensional (2D) water channels that reduce heat loss by decreasing its dimensionality and volume [19]. Furthermore, one-dimensional (1D) water channels separates the water transmission channel from the thermal management structure, resulting in minimal heat radiation, convection, and loss. One such example is the research by Li et al. [20], in which they combined a 1D water channel structure with polystyrene in a jellyfish-like solar evaporator. This evaporator demonstrated an impressive energy conversion efficiency of 87.5% under one sunlight, making it a high desirable option for to improving evaporation rates. The upgrade of evaporator structure from 1 to 3D can significantly increase the overall energy input by enlarging the surface area on a macroscopic scale. This leads to high yield of water evaporation within a certain time frame.

Researchers have extensively studied photothermal materials, which are used for solar energy absorption and conversion. These mainly include semiconductors [21], metal nanoparticles [22], carbon matrix composites [23], and organic conjugated polymers [24]. The photothermal mechanism can be categorized into electron–hole generation and relaxation [6], plasma localized heating, and molecular thermal vibration [25]. However, researchers are still facing challenges such as low photothermal conversion efficiency, insufficient energy management, and poor hydrophilicity in many materials, which affect their effectiveness. The 2D nanomaterial MXene has the advantages of good mechanical stability [26], variety, adjustable thermal radiation loss, and easy control of optical properties [27–31]. However, the high cost, low yield, and the need for additional functionalization during the preparation of MXene limits its application in desalination. The production process of MXene is generally inefficient and results produces in MXene sediments that are considered waste. These sediments consist of unetched Ti_3_AlC_2_ (MAX), unexfoliated multilayered MXene (m-MXene), and a small amount of residual monolayer/oligolayer MXene [32, 33]. Currently, MXene sediments (MS) has been extensively studied in supercapacitors [34], electromagnetic shielding, and smart wearables [35]. However, their potential as photothermal conversion materials for desalination has not yet been explored.

This research addresses above-mentioned issues by introducing 3D aerogel evaporator with vertical multi-dimensional pores, innovatively combining polyvinyl alcohol (PVA)/sodium alginate (SA) and MS. (The obtained is represented as PSMS aerogel.) Additionally, the research discusses the design and assembly of a solar-powered “self-floating evaporation condensation collection device.” The device utilizes cotton swabs as channels for water transmission. Polypropylene floats are used as the evaporator’s support on the sea surface to ensure the normal operation of the evaporator. The photothermal water evaporation system using MS as the main substrate, ensuring strong water absorption, resulting in stable water evaporation over a long period of time. Additionally, the side of the 3D aerogel can be used as the cold evaporation surface during the evaporation process. This allows the energy from the environment can be utilized by taking advantage of the temperature difference with the surrounding environment. As a result, the evaporation rate and energy conversion efficiency of the evaporator can be further enhanced. The evaporator can evaporate up to 3.6 kg m^−2^ h^−1^ at an exposure height of 2.5 cm in one illumination. In a continuous outdoor test for 7 h, it was able to achieve an evaporation rate of 18.37 kg m^−2^. The solar evaporator with an anisotropic vertical channel structure uses directional freezing to support high-speed water transport and bidirectional salt ion diffusion. It can evaporate seawater continuously for 14 days under natural light conditions, without any salt crystals precipitating out of the surface, demonstrating excellent salt resistance. The combination of SA and MS provides superhydrophilicity to the surface of the aerogel evaporator by creating a layer of water film. This water film gives the evaporator excellent oleophobic properties, enabling it to maintain stable evaporation performance even in oily seawater for a long time. Overall, the aerogel evaporator with vertical channels designed in this research has several advantages, including excellent evaporation performance, long-term stability in different environments, and efficient utilization of waste resources.

## Experimental Section

### Preparation of MXene Suspension and MXene Sediments

3.0 g LiF was dissolved in 50 mL HCl solution and stirred at room temperature (Table S1). After stirring for 10 min, 2.5 g of Ti_3_AlC_2_ was slowly added to the above mixture solution and stirred at 40 °C for 24 h. The etched solution was washed 7–9 times with deionized water, and the mixed solution was separated and centrifuged, so that the pH value of the supernatant reached 6, and the inner wall of the centrifuge tube obtained a clay-like sediments. Subsequently, it was dispersed in deionized water (150 mL) and sonicated for 1 h, followed by centrifugation at 3500 rpm for 1 h, and the supernatant poured out was Ti_3_C_2_T_*x*_ MXene (Fig. S1). The “waste” MXene sediments that was usually discarded was left at the inner wall of the centrifuge tube, which are used as photothermal materials for the preparation of PSMS aerogel evaporators (Table S2 and Note S1). To compare the performance, the supernatant Ti_3_C_2_T_*x*_ (MXene) was taken to make PSMX aerogel, and the PVA/SA mixture solution to make PS aerogel.

### Preparation of MXene Sediments Aerogel and Assembly of Evaporator

**Fig. 1:**
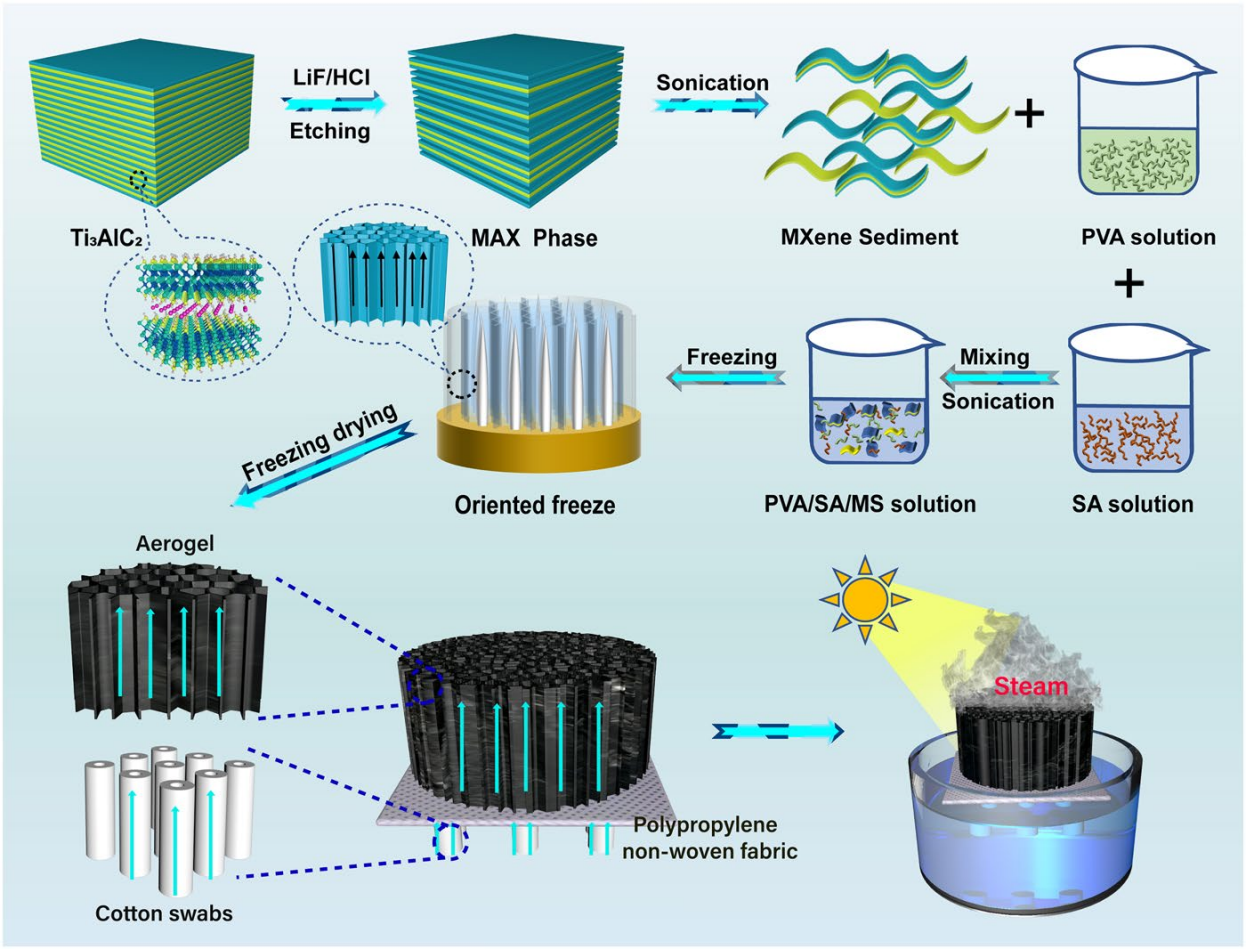
Fabrication process of PSMS aerogel evaporator

5 mL of 1 wt% PVA solution and 15 mL of 1 wt% SA solution were stirred for 5 min at room temperature, 3.6 g of MXene sediments was dispersed into 20 mL of deionized water, then added to the above mixed solution and instilled with 0.4 g γ-glycidyl ether oxypropyltrimethoxysilane as crosslinker (Figs. 1 and S2) (more crosslinker performance test as shown in Note S1). After mixing, magnetic stirring was carried out for 3 h, ultrasonic deaeration was carried out after stirring, and the mixed solution was poured into a custom mold to prepare PVA/SA/MS 3D composite aerogel PSMS-1 by liquid nitrogen directional freezing. PVA/SA composite aerogels, PSMS composite aerogels with 2–4 wt% (PSMS-2, PSMS-3, PSMS-4) and MXene sediments content of 2.6 g (PSMS-5) and 4.6 g (PSMS-6) were prepared according to the above method, respectively.

As shown in Fig. 1, the evaporation device was composed of cotton swabs, polypropylene material, and PSMS aerogel, considering the heat loss in the water transmission process, the water transmission channel and the thermal management structure is separated, and the cotton swab was used as 1D channel to transmit water to the aerogel, and the application of 1D water channel greatly reduces the heat radiation, heat convection, and heat loss of water in the transmission process. Polypropylene material with a density of only 0.9 g cm^3^ was selected for the float, and its nonwoven material can float on the water surface as an aerogel support. In the polypropylene material, a hole with a diameter of 0.5 cm was formed in an array, and the cotton swab was assembled in the hole, and finally, the 3D aerogel was placed on the polypropylene material to contact the cotton swab to ensure that the aerogel can obtain an adequate water supply.

### Characterization

The morphology of the MXene sediments was observed via transmission electron microscopy (TEM, JEM-2100, JEOL, Japan), and the morphology of the composite aerogel was studied by scanning electron microscopy (SEM, EVO18, ZEISS, Germany). The surface chemistry of composite aerogels were characterized using energy dispersive X-ray spectroscopy (EDS). The crystal property and composition were analyzed via X-ray diffraction patterns (XRD, SmarlabSE, Japan) using Cu Kα radiation (*λ* = 1.5406 Å). Fourier transform infrared (FTIR) spectroscopy (Thermo Scientific Nicolet iS50) was used to analyze the functional groups of the evaporator. Elemental analysis of the composite gels was performed using an X-ray photoelectron spectrometer (XPS, Escalab 250 Xi, Thermo Scientific, America). The water contact angle of the aerogels were tested using optical contact angle (OCA 15EC). The optical transmission and reflection spectra of aerogels can be measured by using 300–2500 nm on an ultraviolet–visible-near-infrared spectroscopy (UV-3600, Shimadzu, Japan) with an integrating sphere. The thermal conductivity was tested by a thermal conductivity meter (TC3000E, China) using the hot-wire method. According to Kirchhoff’s law, the absorbance (A) was calculated by reflectivity (R) and transmittance (T), and the formula was A = 1−R−T. The concentration of metal ion was measured by inductively coupled plasma-optical emission spectrometry (ICP-OES, Avio 200, PE) (Table S3).

## Results and Discussion

### Construction and Characterization of PSMS Aerogel

**Fig. 2:**
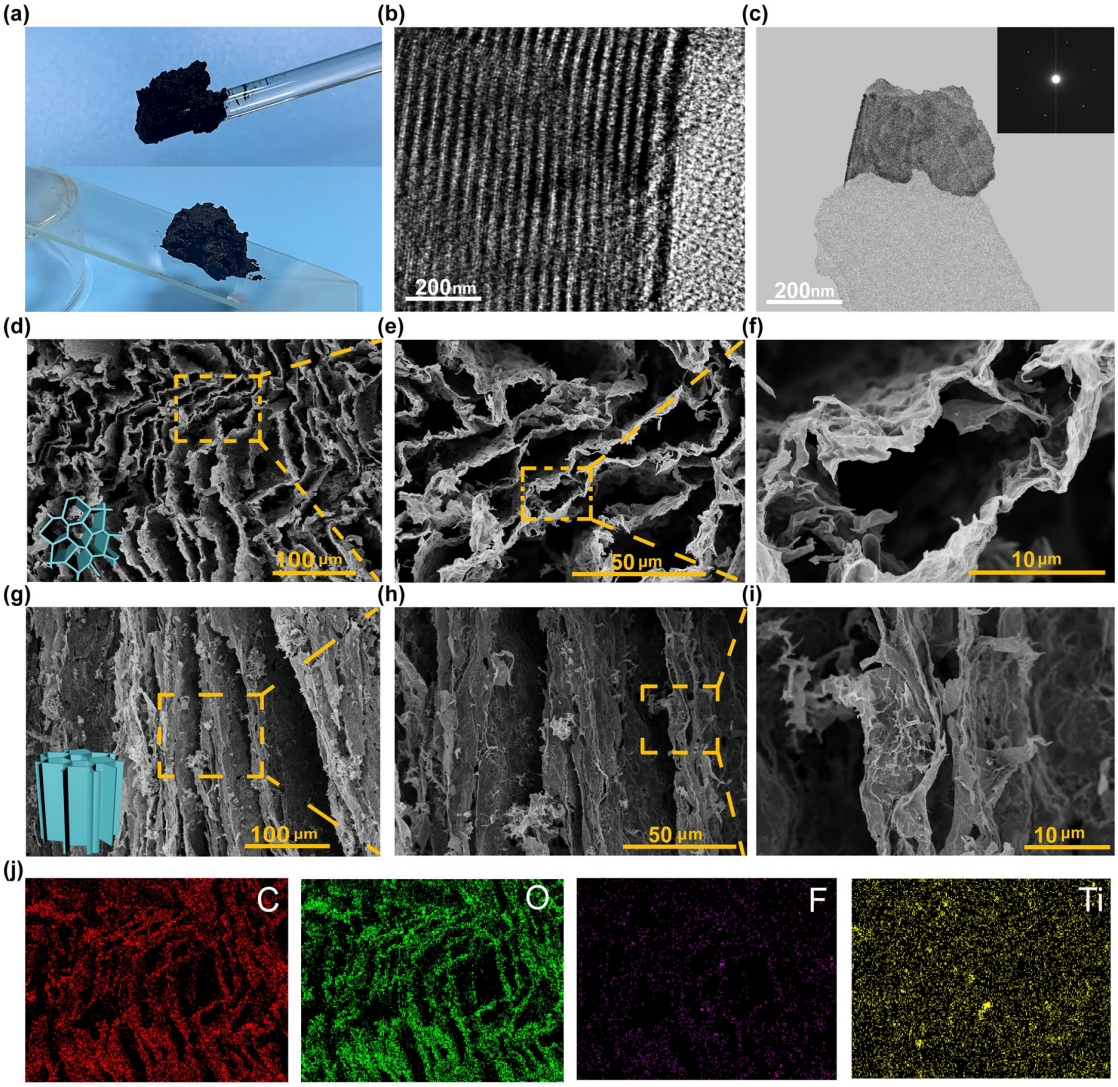
Structural characterization of PSMS aerogel. **a** Shape of collected high-concentration MS (top) ramp after standing for one hour (bottom). **b** TEM diagram of MS (${\text{Ti}}_{3}{\text{Al}}{\text{C}}_{2}$)$.$ **c** TEM image of MS (m-MXene and MXene). **d-f** PSMS aerogel in the transverse direction was observed under SEM. **g-i** PSMS aerogel in the longitudinal direction was observed under SEM. **j** The surface-sectional EDS mapping images of PSMS aerogel

Inspired by the natural wood transpiration process, a 3D evaporator made of PVA, SA, and MS was prepared (Figs. 1 and S1). High-quality Ti_3_C_2_T_x_ MXene nanosheets (supernatant) were separated by etching of the MAX-Ti_3_AlC_2_ phase (Fig. S2a, b) and centrifugation and sonication, leaving a large amount of sediments at the bottom (Figs. 2a and S2c). TEM images show that the MS contained the unetched MAX phase (Fig. 2b) and etched single/few-layered MXene nanosheets (Fig. 2c), demonstrating the coexistence of the MAX phase, m-MXene, and MXene layers. MS with broadband light absorption capacity can effectively increase the sunlight absorption and convert it into heat, making it an ideal photothermal material. Additionally, PVA and SA can improve the mechanical properties and hydrophilicity of aerogels. PSMS aerogels were prepared through a process of directed freezing using liquid nitrogen and followed by freeze drying. During the directed freezing process, a large quantity of ice crystals grew vertically from the base of the mold. The suspension squeezed each other at the ice crystal interface, where water molecules served both as pore-forming and spacer agents, enabling their spontaneously assembly into a porous structure [16]. The vertically arranged porous channels not only mitigated water heat loss during transmission, but also enhanced the efficiency of photothermal conversion. Moreover, they facilitated the rapid evaporation of water vapor at the interface.

The morphology of the prepared PSMS aerogels was examined using SEM. As shown in Fig. 2d, g, in contrast to the previously reported chaotic and interconnected porous structure of aerogels, the PSMS aerogels showed a well-defined hierarchical and organized structure (Fig. S3). Figure 2e, f illustrates aerogels with polygonal pores, featuring average pore diameters ranging from 10 to 70 µm. The channel walls, measuring 0.5–2 µm, were composed of MS, SA, and PVA. The distinctive morphology resulted from the combination of the 2D structure of the MS and the extrusion of the ice crystals during the freezing process. During the freeze drying process, PSMS aerogels developed vertically aligned channels with widths of 30–100 μm and the resulting parallel layered channel walls gave rise to short-range ordered microregions (Fig. 2g–i). This occurrence could be attributed to the unidirectional growth of ice crystals during freezing, where MS sheets were vertically arranged with the direction of ice crystal growth by mechanically shearing the MS disk-like layered liquid crystal phase [36]. The central cross-linked region of the microregion consisted of interlinked porous walls, PVA, and bonding points connecting to SA (Fig. 2i). In particular, these elements formed a continuous vertical microchannel structure, enhancing light absorption and steam release, consequently increasing the evaporation rate. As shown in Fig. 2j, energy dispersive spectroscopy results demonstrated the distribution of C, O, Ti, and F on the aerogel surface. The presence of C and O elements was attributed to MS, PVA, and SA, while Ti and F elements originated only from MS. The consistent distribution of these elements across the entire area confirmed the uniform of dispersion of MS within the porous PVA and SA network structure.

**Fig. 3:**
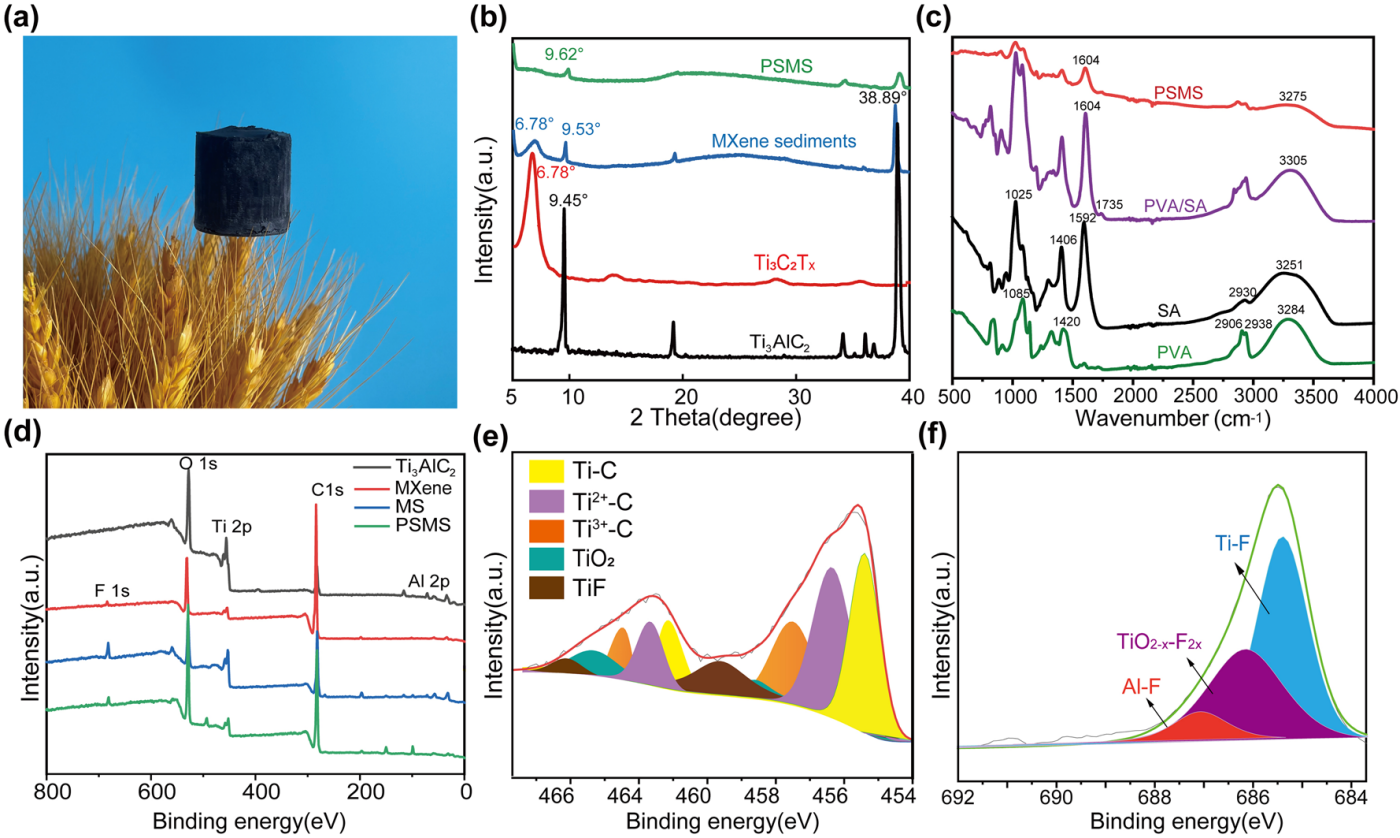
Interactions between different components in PSMS aerogel. **a** PSMS lightweight diagram. **b** XRD test plots of Ti_3_AlC_2_, MS, Ti_3_C_2_T_x_ MXene, and PSMS aerogel. **c** FTIR spectrum of PVA, SA, PS and PSMS. **d** Full XPS spectra of Ti_3_AlC_2_, MXene, MS and PSMS aerogels. **e, f** Ti 2*p* XPS patterns (**e**) and F 1*s* XPS patterns (**f**) of MS and PSMS aerogel

The ultra-light nature of PSMS aerogel is shown in Fig. 3a, where a 2 cm diameter, 2.5 cm high aerogel rests on wheat without causing deformation. Additionally, the weight comparison between the dry and wet states of the aerogel (Fig. S4) further underscores the excellent properties of PSMS aerogel, demonstrating low density and high porosity. The XRD patterns of the Ti_3_AlC_2_, Ti_3_C_2_T_*x*_ MXene, MS, and PSMS aerogel are illustrated in Fig. 2b. The Ti_3_AlC_2_ exhibited two strong characteristic peaks at 9.45° and 38.89°. After etching, the characteristic peak of Ti_3_AlC_2_ shifted from 9.45° to 6.78°, and the 38.89° characteristic peak nearly disappeared. This observation suggests that the Al layer in the Ti_3_AlC_2_ underwent etching, resulting in the formation of Ti_3_C_2_T_x_ MXene. After successfully preparing of MXene, the remaining sediments comprised MS, exhibiting the characteristic peaks at 6.78°, 9.53°, and 39°. This further verified the presence of unetched MAX phase, incompletely exfoliated multilayers of Ti_3_C_2_T_*x*_, and residual MXene in the sediments. Upon compounding the MS with PVA and SA, the PSMS aerogel was prepared. Subsequently, after the addition of SA and PVA, the characteristic peak at 6.78° vanished, while the characteristic peak intensity at 39° diminished, indicating the uniform recombination of MS within the aerogel.

As shown in Fig. 3c, the peaks of PS aerogel were slightly shifted to higher bands (3305 and 1604 cm^−1^) compared to the peaks of PVA (–OH 3284 cm^−1^) and SA (C–O–C 1592 cm^−1^), suggesting intermolecular and intramolecular hydrogen bonding interactions between PVA and SA. PS aerogel corresponds to the stretching vibration of the –OH group at 3305 cm^−1^, and the addition of MS shifted the hydroxyl peak of PSMS aerogel to the low-wave number region by 30 cm^−1^, which suggests that the polar terminal groups (–F, –O, and –OH) present on MXene sediments can establish intermolecular hydrogen bonds with the abundant –OH groups on PS aerogel [37]. Meanwhile, the PSMS aerogel has weakened characteristic peaks at 3000–3600 cm^−1^, indicating that the interaction between terminal hydroxyl group and PVA after the addition of MS led to the weakening of the interaction between the PVA chain molecules, and a decrease in the crystallinity of PVA in PSMS aerogel could possibly contribute to the improvement of porosity and water absorption capacity.

The full XPS spectra of Ti_3_AlC_2_, MXene, MS and PSMS aerogels are shown in Fig. 3d. Ti_3_AlC_2_ and MXene lack the F and Al elements, respectively, whereas MS and PSMS contain both elements, indicating that MS possesses both unetched Ti_3_AlC_2_ and MXene. Also the presence of Si element in the PSMS aerogel indicates a uniform distribution of the crosslinker. To investigate the elemental composition and elemental chemical state of the MS, the high-resolution spectra were further analyzed. The Ti 2*p* spectra of MXene sediments (Fig. 2e) deconvoluted five peaks at 455.4 (461.3), 457.4 (432.2), 458.5 (464.3), 459.5 (464.6), and 460.5 (465.8) eV belonging to the Ti-C, Ti(II), Ti(III), TiO_2−*x*_F_2*x*_, and TiF_3_ [38]. In addition, the major F 1*s* XPS peaks in Fig. 3f were fitted to three peaks, Ti-F (685.3 eV), TiO_2−*x*_F_2*x*_ (686.1 eV), and Al–F (683.3 eV).

### Hydrophilicity, Light Absorption, and Thermal Management Performance of PSMS Aerogel

**Fig. 4:**
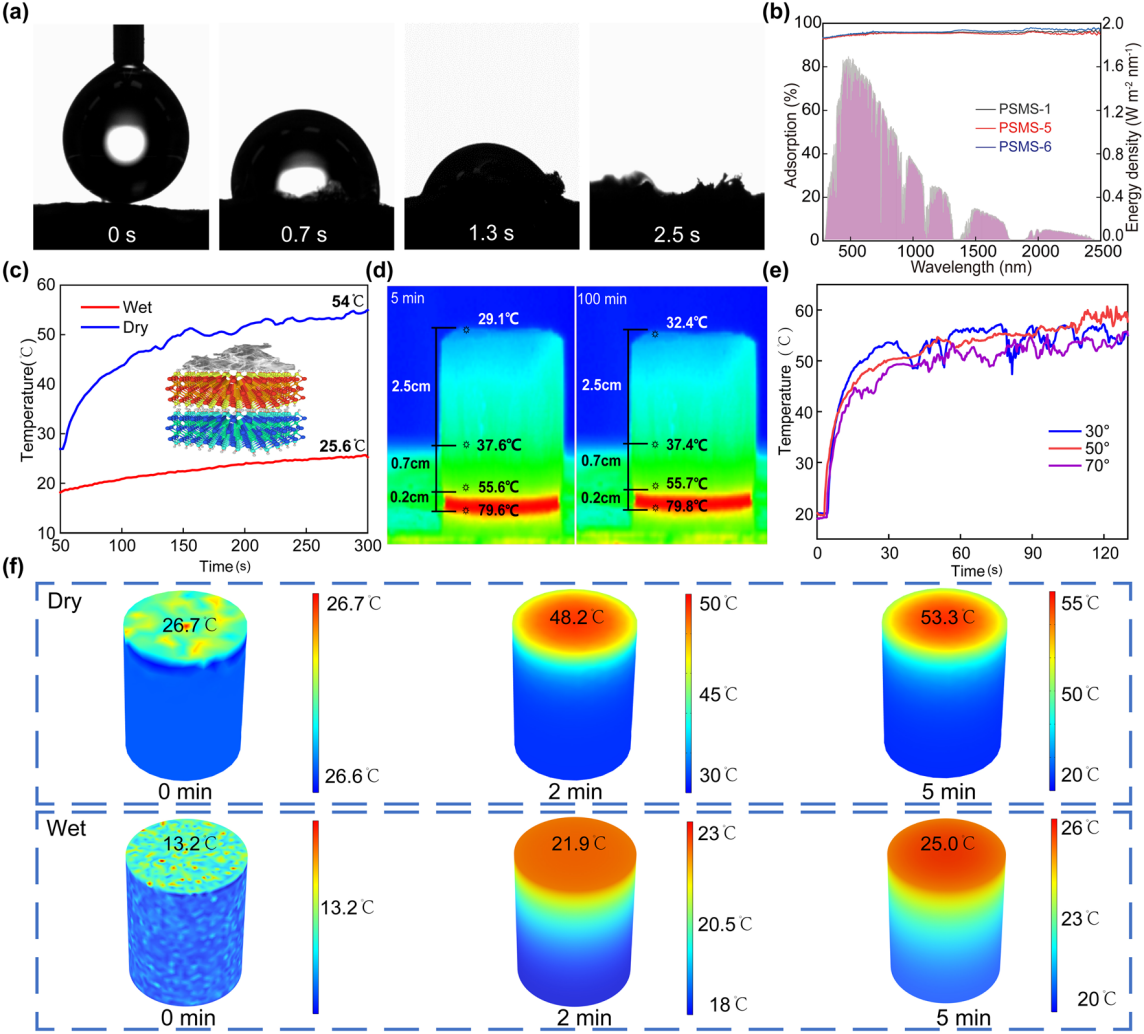
Hydrophilicity, light absorption, and thermal management performance of PSMS aerogel. **a** Wettability of the PSMS aerogel. **b** Absorption spectra of PSMS-1, PSMS-5, PSMS-6 aerogels. **c** Temperature difference between PSMS-1 aerogel in wet and dry state under 1 sunlight irradiation. **d** Infrared image of PSMS-1 aerogel in dry state on a hot plate. **e** PSMS-1 aerogel temperature change of the evaporator at different angles of incidence. **f** COMSOL simulation calculating the surface temperature of PSMS-1 aerogel in the dry and wet states for rapid warming within 5 min

During the evaporation process, the wettability of the material and efficient water transport are crucial for generating steam at the solar-driven interface. In Fig. 4a, the PSMS aerogel absorbed a water droplet completely within just 2.5 s. The rapid absorption can be attributed to the abundant carboxyl and hydroxyl groups present in SA. Additionally, the hydrogen bonding between the SA and PVA significantly enhanced the water absorption performance of the evaporator. Moreover, MS showed good hydrophilicity. The loosely arranged vertical pores in the aerogel contribute to a better core-absorption effect, enhancing the water transport capacity of aerogels. To emphasize the significance of the vertical channel structure in water transport, an aerogel with disordered channels was fabricated for comparative analysis. Based on the height of colored water transport within 8 s shown in Fig. S5, the aerogel with liquid nitrogen-frozen vertical channel exhibited considerably greater height compared to the aerogel with disordered channels frozen in a refrigerator. Consequently, this design allows for the rapid water transport to the evaporator surface, ensuring efficient water supply throughout the evaporation of the PSMS aerogel.

To assess the light absorption capacity of PSMS aerogels, the researchers compared the absorption spectra of aerogels prepared with varying MS contents in the ultraviolet–visible-near-infrared range (Fig. 4b). The PSMS aerogel showed lower transmittance (~ 0%) and reflectivity within the of 300–2500 nm. Notably, the light absorption rate of PSMS-5 aerogel evaporator with the lowest MS content achieved 96%, while the PSMS-6 aerogel evaporator, featuring the highest MS content, showed an even higher light absorption rate of 98%. The outstanding light absorption of PSMS aerogel results from the combination of strong absorption effects of MS and the presence of vertical porous channels. Significantly, the MS on the surface of aerogel can either induce light reflection or transmission while efficiently absorbing the internal light into the vertically arranged channels [39].

The effectiveness of photothermal conversion is essential for the evaporation performance of solar evaporators. Consequently, the photothermal conversion performance of aerogels is tested and thoroughly investigated. The excellent sunlight absorption and photothermal conversion capabilities of the MS enable it to generate a substantial amount of heat under sunlight. Simultaneously, the vertically porous structure achieved through directional freezing better confined the generated heat to the evaporator’s surface, thereby further improving the efficiency of photothermal conversion [40]. The surface temperature of the aerogel was measured in dry and wet states under 1 kW m^−2^ of illumination using an infrared thermal imager (Figs. 4c and S6). Apparently, the surface temperatures of the aerogels increased rapidly, and the dry and wet too aerogels reached 54 and 25.6 °C after 300 s, and finally stabilized at 68.5 and 26.7 °C, respectively. This indicates that the evaporator has excellent light absorption performance, and the MS-induced plasma localized heating effect also contributes to its good photothermal conversion performance. Essentially, the evaporator operated in the wet state during the evaporation process, as shown in Fig. S6 (right). The side temperature of PSMS aerogel in the wet state was only 16.2 °C, significantly lower than the ambient temperature of 22.6 °C. Consequently, the process of water transportation from bottom to top not only mitigated the influence of heat loss, but also absorbed heat from the environment [41, 42]. These results indicate that the PSMS aerogel exhibits excellent thermal management performance.

In the dry state, the transversal and longitudinal thermal conductivities (more detail discussion as shown in S1.1) of aerogel were 0.0404 and 0.0445 W m^−1^ K^−1^, respectively. However, after wetting, it increased to 0.1786 and 0.1829 W m^−1^ K^−1^, respectively (Fig. S7). This rise in thermal conductivity is attributed to the shift in the medium from air (0.026 W m^−1^ K^−1^) to water (0.6 W m^−1^ K^−1^) within the pores. The lower thermal conductivity signifies that the aerogel developed in this research boasts excellent thermal insulation, promoting the establishment of “localized heating” during the evaporation process [43, 44]. As shown in Fig. 4d, the evaporator was positioned on a hot stage at 80 °C for 100 min. Over time, the temperature at the top of the aerogel remained at approximately 32 °C. The temperature difference between the bottom and top of the aerogel was around 48 °C, indicating the evaporator’s good thermal insulation and excellent thermal localization (Fig. S8). For practical desalination applications, considering the sun’s changing position over time, studying the absorption capacity of solar irradiation at various incidence angles becomes essential in the preparation of efficient solar evaporators. Analyzing the surface temperature infrared images of the evaporator at different angles of incidence reveals that as the angle between of the aerogel’s vertical direction and the direction of sunlight incidence increases from 30 to 70 °C, the surface temperature of the evaporator undergoes minimal change, stabilizing between 50 and 60 °C (Figs. 4e and S9). At large angle of incidence, the surface temperature of the composite aerogel exhibited a slight decrease. This phenomenon is attributed to the gradual reduction of the illuminated projection area, indicating that the PSMS aerogel showed low dependence on the illumination direction and good photothermal conversion stability. Further, to verify the accuracy of this heat transfer model, the simulated warming of the dry and wet PSMS-1 aerogel surface was simulated within 5 min using COMSOL Multiphysics software (more detail discussion as shown in S1.2). The PSMS aerogels were rapidly warmed up to 53.3 and 25 °C for simulations in the dry and wet states. The difference from the actual temperature was 0.7 and 0.6 °C, respectively (Fig. 4c, f). The numerical model exhibited good agreement with the experimental data, confirming the accuracy of this heat transfer model (Tables S4, S5 and Notes S2, S3). The aforementioned results indicate that the PSMS aerogel in this research demonstrates excellent thermal localization and thermal barrier performance. These characteristics are expected to minimize the heat loss during the evaporation process, consequently enhancing the utilization rate of energy for photothermal conversion.

### Evaporation Performance of PSMS Aerogel Evaporator

**Fig. 5:**
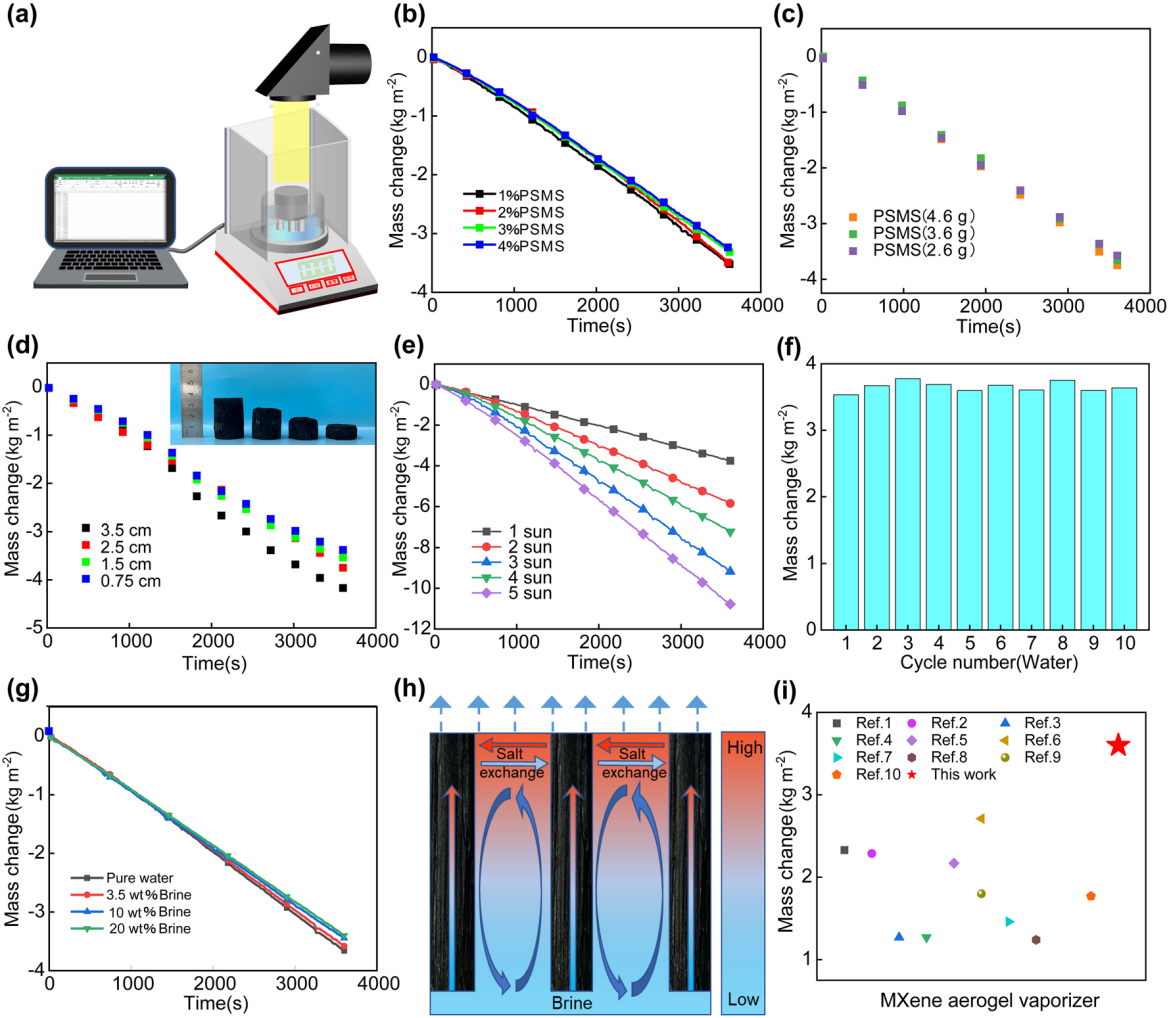
Evaporation performance of PSMS aerogel evaporator. **a** Schematic diagram of solar evaporation experimental device. **b** Changes in evaporated water quality of evaporators with different concentrations of PSMS aerogel evaporator (2.5 cm).** c** Different MS content PSMS aerogels (2.5 cm) change of evaporated water quality of evaporator. **d** Changes in the quality of evaporated water in PSMS aerogel evaporators at different heights. **e** Changes in the quality of evaporated water in the PSMS-1 aerogel evaporator under 1–5 solar conditions. **f** The rate of 10 water evaporations under 1 sunlight. **g** Changes in the quality of evaporated water at different salt concentrations. **h** Schematic diagram of salt-water exchange. **i** Comparison of evaporation rates of different MXene photothermal material evaporators

In the PSMS aerogel evaporator, the evaporation rate is influenced by various factors, and the real-time mass changes are measured using a computer-connected electronic balance (accuracy 0.1 mg) (Fig. 5a). As shown in Fig. 5b, it demonstrated the water mass change for evaporator with the same MS content but different concentrations of aerogels during evaporation under 1 sun illumination. The evaporation rates for 4%, 3%, 2%, and 1% evaporators were measured 3.2, 3.35, 3.5, and 3.6 kg m^−2^ h^−1^ (more detailed calculation as shown in S1.3), respectively. As the aerogel concentration decreases, there is a corresponding increase in the evaporation rate. This occurs because with a lower aerogel concentration, the water content in the solution rises. During the directed freezing process with liquid nitrogen, ice crystals grow vertically from bottom to top. The increased water content acts as spacers, leading to larger the pore size within the aerogel evaporator after freeze drying. These enlarged pores create favorable conditions for water transmission and heat radiation conduction inside the evaporator, contributing to the increase in the evaporation volume [45]. Next, the variations in water mass were examined for evaporators with verifying MS contents under 1 sun illumination. As observed in Fig. 4c, the evaporation rates for evaporators with 2.6, 3.6, and 4.6 g under simulated solar irradiation were 3.4, 3.6, and 3.9 kg m^−2^ h^−1^, respectively. These results suggest that higher MS content correlates with more effective evaporation. This is attributed to the abundance of active groups and the high specific surface area on the surface of MS. These features allow MS to efficiently absorb sunlight and convert it into heat, thereby enhancing the thermal localization effect on the evaporator surface. Consequently, the light-heat conversion ability is strengthened, leading to a higher evaporation rate. According to Fig. 5c, it is evident that the relationship between evaporation rate and MS content does not increase linearly. To optimize manufacturing costs, the aerogel with MS content of 3.6 g is selected as the subsequent experimental concentration.

For the 3D evaporator, the side area also played a crucial role in determining the evaporation rate. PSMS aerogel evaporators of different heights (0.75, 1.5, 2.5, and 3.5 cm) (Fig. S12) were designed at 1 wt% concentration and 3.6 g MS content. The water evaporation rate under 1 sun vertical irradiation was measured. As illustrated in Fig. 5d, the evaporation rate increased from 3.1 to 4.0 kg m^−2^ h^−1^ with an increasing height. This is primarily due to the additional water–air interface created by the 3D evaporators with sidewall area while maintaining a consistent projection area. The evaporator side surface evaporates in a wet state causing its temperature to be lower than the ambient temperature. This facilitates the acquiring of additional energy from the surrounding environment (PSMS-1–0.213 W, more detailed calculation as shown in Note S4) [46]. Simultaneously, the side surface of the PSMS aerogel evaporator can effectively decrease the average temperature of the solar evaporation surface, considerably minimizing heat loss from the top surface flow and heat radiation. To save manufacturing costs, evaporators with a height of 2.5 cm were selected to investigate the water evaporation rate of PSMS aerogels under different light intensities. As shown in Fig. 5e, the evaporation rates of PSMS-1 aerogels under 1, 2, 3, 4, and 5 sun illumination were 3.6, 5.9, 7.8, 9.7, and 11 kg m^−2^ h^−1^, respectively. The experimental results demonstrate that the evaporation rate increases with the rise of light intensity. The evaporators exhibit stable and excellent evaporation rate even under high sunlight irradiation. According to formula (1–3), we calculate the evaporation solar-to-vapor energy conversion efficiency (*η*) of PSMS-1 [47–49]:

1 $$\eta =\frac{{V}_{n}\left({H}_{lv}+Q\right)}{{E}_{i}}$$

2 $$  {\text{H}}_{{{\text{lv}}}}  = 1.91846 \times 10^{6}  \times \left( {\frac{{{\text{T}}_{{\text{a}}} }}{{{\text{T}}_{{\text{a}}}  - 33.91}}} \right)  $$

3 $$ {\text{Q}} = {\text{c}} \times \left( {T_{a}  - T_{i} } \right)  $$

where *V*_n_ is the net evaporation rate, *H*_lv_ is the latent heat of the evaporation (J kg^−1^), Q is the sensible heat of water, *E*_i_ is the energy input by the incident light (kJ m^−2^ h^−1^), c is the specific heat capacity of water (4.2 J g^−1^ K^−1^), *T*_a_ is the temperature (K) of evaporation, and *T*_i_ is the initial temperature (K) of water (detailed calculation as shown in Note S5). The solar-to-vapor energy conversion efficiency of PSMS-1 was calculated to be 189%, revealing a superior solar-to-vapor conversion efficiency. The durability and long-term stability of the PSMS aerogel evaporator were tested. Clearly, as-prepared PSMS-1 aerogel evaporators show excellent recoverability and strong durability. The evaporation rate and solar-to-vapor energy conversion efficiency remained largely stable at about 3.6 kg m^−2^ h^−1^ and 189% even after exposure to 10 light cycles (Fig. 5f).

In the application of seawater desalination, the salt deposition layer not only amplifies sunlight reflection, impeded water transmission speed, and diminishes the evaporation rate, but it also considerably impacts the service life of the evaporator. Further, salt resistance of the evaporator is crucial in the application process, serving as a key for the long-term, efficient, and stable operation of the evaporator. There is a need for the design of new solar evaporators with salt resistance to advance interfacial solar desalination evaporation system technology. To investigate the salt resistance of the evaporator, PSMS-1 aerogel evaporator was selected to test the evaporation rate at different salt concentrations. As shown in Fig. 5g, h, the evaporation rate of PSMS-1 aerogel evaporator in pure water, 3.5 and 20 wt% brine can be maintained at about 3.5 kg m^−2^ h^−1^. Under one sunlight illumination, the evaporator underwent 10 times cycles of water evaporation in 3.5 and 20 wt% brine (Fig. S13). The average evaporation rate and efficiency of 3.5 wt% brine for 10 evaporations were 3.6 kg m^−2^ h^−1^ and 188%, respectively. The average evaporation rate and solar-to-vapor energy conversion efficiency of 20 wt% brine for 10 evaporations were 3.43 kg m^−2^ h^−1^ and 179%, respectively. These results show that the evaporator can remain stable for long periods of time without increasing salinity, even under extreme conditions of high salinity for long periods of time. Additionally, the evaporator was immersed in natural seawater (Yellow Sea, Qingdao, China) under ambient sunlight for 14 days (May 2023; Qingdao, China, Fig. S14). Evaporation rates before and after 14 days were 3.62 and 3.59 kg m^−2^ h^−1^, respectively, and no salt crystallization was observed on the evaporator surface. The above results indicate that the evaporator has a stable and excellent evaporation rate and salt resistance even under prolonged operating conditions. The salt resistance of the evaporator can be attributed to the vertically ordered microscopic channel structure formed through directional freezing. This structure allows the PSMS aerogel to function as a supporting matrix, facilitating continuous water delivery to the evaporator surface through capillary action. Simultaneously, the anisotropic structure enhances ion exchange capacity between the channels. The liquid on the evaporator surface establishes a concentration gradient due to efficient photothermal conversion, gradually reaching saturation through effective evaporation. At this stage, the dissolved salt ions in the saturated solution gradually flow back into the brine along the water transport channels in PSMS aerogel evaporator (Fig. 5h). This movement may be driven by the chemical potential difference between the saturated solution and the brine. The entire system can work together to attain the equilibrium of high-concentration salt solutions and inhibit the precipitation of salt crystals [40]. Compared with PSMX aerogel, the PSMS aerogel evaporator surface temperature and evaporation rate were only 1.9 °C and 0.2 kg m^−2^ h^−1^ lower (Figs. S6 and S15), respectively. Probably the poor photothermal properties of the MAX phase and the multilayer MXene (m-MXene) in the PSMS aerogel resulted in a slightly lower surface temperature. But in comparison (Fig. 5i and Table S6), the evaporation rate of the 3D PSMS aerogel evaporator designed in this research is notably higher than that of majority of 3D evaporators reported to date [50–59]. This significant improvement can be attributed to its well-integrated structure, excellent thermal confinement, and timely upward water convection. It also validates the reliability and practicality of utilizing the “waste” material MS for the preparation of seawater desalination evaporators.

### Application of PSMS Aerogel Evaporator

**Fig. 6:**
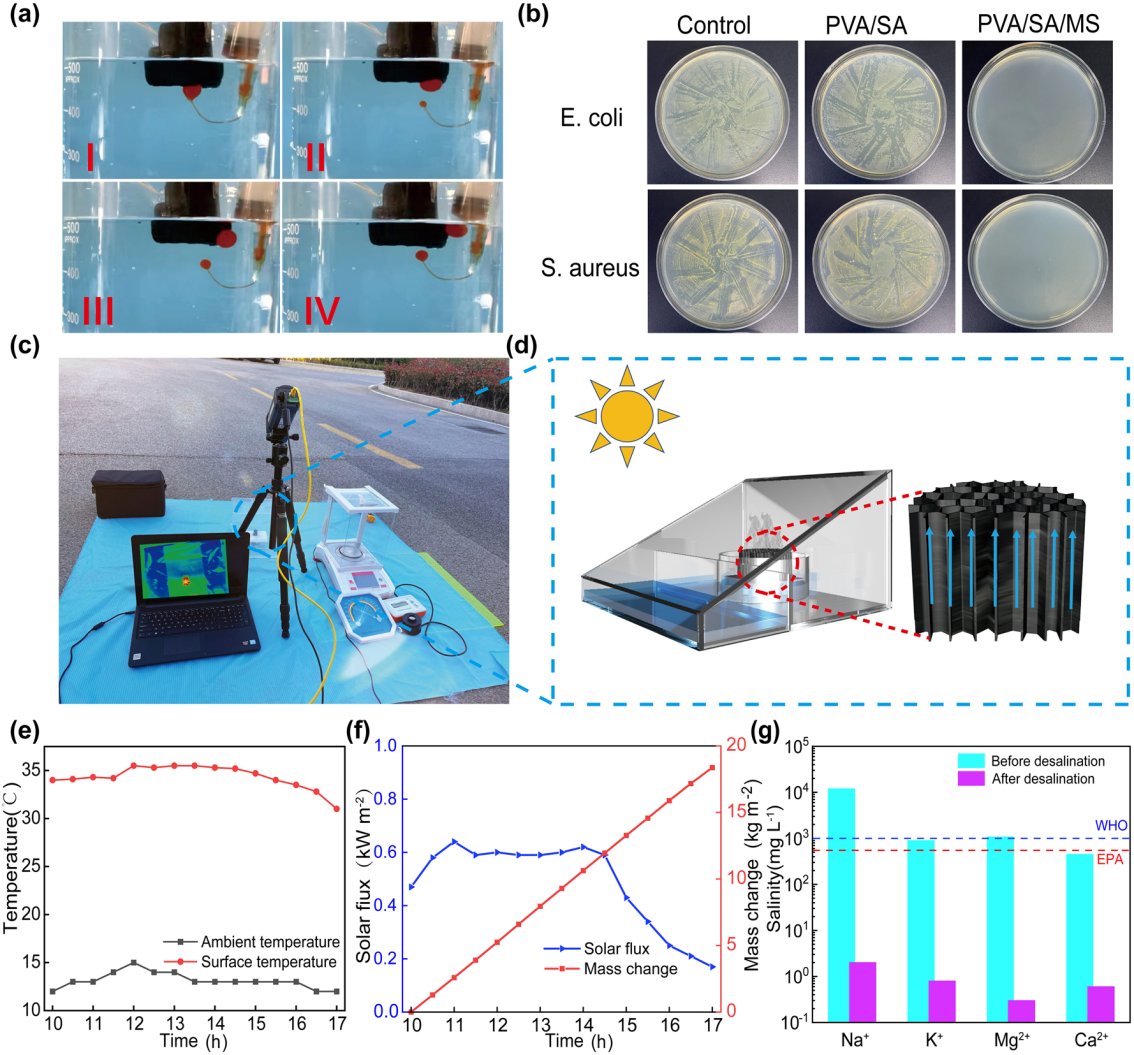
Application of PSMS aerogel evaporator. **a** PSMS-1 aerogel macroscopic diagram of underwater oil pollution escape of evaporator. **b** Antimicrobial properties of the PSMS-1 aerogel. **c** Digital image of the “Evaporation–Condensation-Collection“ device of the outdoor. **d** Schematic diagram of evaporation device. **e** Outdoor ambient temperature, PSMS-1 aerogel evaporator surface temperature change. **f** Changes in sunlight intensity and water evaporation quality. **g** Changes in metal ion concentration before and after seawater purification

The presence of organic pollutants such as oil, in seawater, is unavoidable. In such cases, oil contamination may adhere to the surface of the evaporator, partially obstructing the seawater transmission channels and thereby reducing the actual evaporation performance [60]. To simulate oil contamination in seawater, vegetable oil was employed, revealing an underwater contact angle with the evaporator of 148° (Fig. S16a), indicating excellent underwater superoleophobic properties. The PSMS aerogel evaporator surface was quickly sprayed by vegetable oil, which immediately escaped without leaving any oil droplets on the aerogels’ surface (Fig. 6a), thus further demonstrating the excellent oil fouling resistance of the PSMS aerogel evaporator. The superoleophobic performance of the evaporator is attributed to the superhydrophilic properties conferred by SA and MS in PSMS aerogels, forming a highly stable water film layer on the surface (Fig. S16b). This protective layer prevents oil from infiltrating the aerogel, affirming the evaporator’s capability to maintain stable evaporation performance even in contaminated seawater. In addition, the PSMS aerogel evaporation rates were tested in extreme environments using HCl at pH = 1 (Fig. S17a), NaOH at pH = 12 (Fig. S18a), and emulsified oil–water mixtures (Fig. S19a) to simulate domestic and industrial wastewater discharged into seawater. The evaporation rates of acidic and alkaline corrosive solutions are 3.2 kg m^−1^ h^−1^ (Fig. S17b) and 3.3 kg m^−1^ h^−1^ (Fig. S18b), respectively, while the emulsion evaporation rate is comparable to that of pure water at 3.6 kg m^−1^ h^−1^ (Fig. S19c). Following purification, no oil droplets were observed in the condensate water (Fig. S19b). These findings demonstrate that PSMS aerogel evaporator can maintain stable evaporation performance and achieve efficient water purification even under challenging seawater conditions. To avoid the risk of epidemics caused by bacteria and other microorganisms, representative strains of Staphylococcus aureus and Escherichia coli were selected to test the antimicrobial effect of the evaporator (more detail discussion as shown in S1.4). As shown in Fig. 6b, minimal bacterial growth was observed on the solid agar plates of PSMS aerogel evaporator, indicating its superior antimicrobial activity, with antimicrobial rates exceeding 99.9% in all cases. This phenomenon arises from the hydrophilic and negatively charged surface properties of the monolayer and multilayer nanosheets in MS, which enhance the interaction with cell membranes. The sharp edges of the nanosheets contribute to damaging the bacterial cell membranes, leading to bacteria death through the leakage of substantial of cytoplasmic components. Hence, MS can effectively prevent the evaporator from contamination and corrosion by microorganisms during prolonged use.

To further assess the practical application of the PSMS aerogel evaporator in desalination under natural light, a condensation device, created using transparent acrylic plates for collecting purified water, was employed. The seawater evaporation efficiency of the PSMS aerogel evaporator with a height of 2.5 cm was tested outdoors under natural sunlight. The surface temperature of the evaporator was recorded with an infrared camera (Fig. 6c). The condensing unit featured a roof-type slope design, incorporating an acrylic plate as a middle barrier. On one side, the evaporation device and actual seawater were positioned. During the evaporator’s operation, the steam would condense into water droplets on the top and flow into the other side of the barrier plate for collection (Fig. 6d). The PSMS aerogel evaporator was positioned in the condensing device from 10:00 to 18:00, and the test recorded variations in ambient temperature, evaporator surface temperature, sunlight intensity, and water quality. In Fig. 5f, it can be observed that the evaporator surface temperature fluctuated with the intensity of sunlight. The sunlight intensity gradually increased from 0.47 kW m^−2^ at 10 a.m., reaching a maximum sunlight intensity from 12:00 to 14:00 of about 0.6 kW m^−2^. The surface temperature of the evaporator rose with the intensity of sunlight, stabilizing at approximately 35 °C (Fig. 6e), considerably higher than the actual ambient temperature. Between 10:00 and 17:00, the PSMS aerogel evaporator condensing unit collected a total of 18.37 kg m^−2^ of fresh water (Fig. 6f). The evaporation rates were not as high as those measured in the laboratory, primarily due to the lower sunlight intensity. The water quality was directly assessed through a straightforward resistance test (Fig. S20), where the electrode distance was fixed and the ohmic value was measured using a multimeter. The resistance values for real seawater, domestic water, and purified water were 0.07, 5.12, and 1.07 MΩ, respectively, demonstrating the excellent ability of the PSMS aerogel evaporator to purify seawater.

Natural seawater (Yellow Sea, Qingdao, China) underwent evaporation using a PSMS aerogel evaporator, followed by collection using a condensation device. Subsequently, Ca^2+^, K^+^, Mg^2+^, and Na^+^ concentrations were tested before and after seawater purification using inductively coupled plasma-optical emission spectrometry (ICP-OES). As shown in Fig. 6g, the concentration of metal ions after purification had decreased, meeting the standard set by the World Health Organization (WHO) and the US Environmental Protection Agency (EPA) for the salinity content of drinking water. The result demonstrates that the purification system employing the PSMS aerogel evaporator can yield clean fresh water from seawater using the photothermal interface evaporation technology. This provides a new and effective way to address the issue of shortage of freshwater resources.

## Conclusion

In conclusion, addressing the issue of resource wastage during the preparation of MXene, this research introduces the design and development of a 3D PSMS aerogel evaporator using MXene sediments as the raw material, complemented by the assembly of a condensate collection device. Excellent photothermal conversion efficiency, evaporation rate, salt resistance, and resistance to biological/oil/special environments are achieved in practical desalination. The ordered porous structure resulting from directional freezing not only facilitates water transport, but also enhances light absorption within the channels, maximize the efficiency of photothermal conversion. The 3D structure is engineered to enhance vapor diffusion at the water–air interface, with side temperatures kept lower than the ambient temperature to absorb additional heat from the environment and increase dark evaporation from the sides. The evaporator achieves an evaporation rate of 3.6 kg m^−2^ h^−1^ under 1 solar irradiation, and the condensation collection device attains a high rate of 18.37 kg m^−2^ for 7 consecutive hours outdoors. Meanwhile, the unique anisotropic structure enables the generation of a chemical potential difference to drive between the saturated solution and brine, achieving equilibrium in the highly concentrated salt solution. This promotes the convection and diffusion, while inhibiting the precipitation of salt crystals. Additionally, the water film layer on the surface of the PSMS aerogel prevents oil from entering the aerogel. The evaporator also exhibits excellent antimicrobial properties and can maintain stable evaporation performance in the extreme environment of seawater. The combination of a self-floating device with a condensation collection device addresses the collection challenge for desalination, and the purified water collected by condensation meets the standards of WHO and EPA. This proves that the PSMS aerogel evaporator is a new method to sustainably develop salt-resistant and highly efficient freshwater resource recovery. Using MS as a photothermal material maximize the potential of the MXene raw material. The development of MS-based evaporators will be expanded in the field of interfacial solar desalination evaporators in the future.

## Supplementary Information

Below is the link to the electronic supplementary material.

Supplementary file 1 (DOCX 2302 kb)
