# Supplementary material for: MXene Sediment-Based Poly(vinyl alcohol)/Sodium Alginate Aerogel Evaporator with Vertically Aligned Channels for Highly Efficient Solar Steam Generation
Source: Nanomicro Lett. 2024 Jun 17;16:220. doi: 10.1007/s40820-024-01433-1 (PMC11183014; doi:10.1007/s40820-024-01433-1)
Supplement: Supplementary file 1 — (DOCX 2302 kb) [file 40820_2024_1433_MOESM1_ESM.docx]

Supporting Information for

**MXene Sediment-based Poly(vinyl alcohol)/Sodium Alginate Aerogel Evaporator with Vertically Aligned Channels for Highly Efficient Solar Steam Generation**

Tian Wang^1,#^, Meng Li^1,#^, Hongxing Xu^1,#^, Xiao Wang^1^, Mingshu Jia^1^, Xianguang Hou^1^, Shuai Gao^1^, Qingman Liu^1^, Qihang Yang^4^, Mingwei Tian^1^, Lijun Qu^1^, Zhenhua Song^2,*^, Xiaohu Wu^3,*^, Lili Wang^1,*^, Xiansheng Zhang^1,*^

^1^ Shandong Key Laboratory of Medical and Health Textile Materials, College of Textiles and Clothing, State Key Laboratory of Bio-Fibers and Eco-Textiles, Research Center for Intelligent and Wearable Technology, Qingdao University, Qingdao 266071, P. R. China

^2^ Department of Pharmacology, Qingdao University School of Pharmacy, Qingdao University, Qingdao 266071, P. R. China

^3^ Shandong Institute of Advanced Technology, Jinan 250100, P. R. China

^4^ College of Electromechanical Engineering, Qingdao University of Science and Technology, Qingdao, 266061, P. R. China

^#^ Tian Wang, Meng Li, and Hongxing Xu contributed equally to this work.

*Correspondence authors: E-mail: songzh@qdu.edu.cn (Zhenhua Song); xiaohu.wu@iat.cn (Xiaohu Wu); llwang@qdu.edu.cn (Lili Wang); xshzhang@qdu.edu.cn (Xiansheng Zhang)

**S1 Supporting Experimental section**

**S1.1 Thermal conductivity test methods and principles**

Dry and wet PSMS aerogels were measured using a thermal conductivity meter (TC3000E, China) through the hot-wire method. The hot-wire method is based on the principle of placing a metal wire as the heat source of the test system inside a specimen with a uniform initial temperature distribution, and then applying a voltage to the metal wire to increase its temperature. Based on the wire heat source temperature rise can be obtained the thermal conductivity of the medium under test. The basic working equations is as follows:

$\lambda=\frac{q}{4\pi A}=\frac{q}{4\pi\left( d\Delta T_{id}/d\ln t \right)}$  (S1)

**S1.2 COMSOL Multiphysics simulation of temperature distribution in PSMS-1**

In this study, finite element simulations were performed using the Darcy flow and heat transfer module of the COMSOL Multiphysics software. The flow field solved by Darcy's law was coupled with the heat transfer equations to simulate the flow and heat transfer processes inside the PSMS-1 solar evaporator within five minutes. The governing equations for Darcy flow is as follows：

$\nabla\cdot\left（ \rho_{f}u \right）=Q_{m}$ (S2)

$u=-\frac{k}{\mu}\nabla p$ (S3)

where u is the Darcy’s velocity, $p$ is the pressure. $k$ is the permeability of the evaporator, $Q_{m}$ is the evaporation mass flow rate, $\rho$ and $\mu$ are the density and viscosity of the water. The equivalent heat transfer model of porous media was used to describe the heat transfer process in solar evaporator:

$\left( \rho C_{p} \right){}_{e\mathrm{ff}}\frac{\partial T}{\partial t}+\rho_{f}C_{P,f}u\nabla T+\nabla q=Q$ (S4)

$q=-k_{e\mathrm{ff}}\nabla T$ (S5)

$\left( \rho C_{p} \right){}_{e\mathrm{ff}}=\theta_{s}\rho_{s}C_{p,s}+\left( 1-\theta_{s} \right)\rho_{f}C_{p,f}$ (S6)

$k_{e\mathrm{ff}}=\theta_{s}k_{s}+(1-\theta_{s})k_{f}$ (S7)

where $Q$ is the heat source; $\left( \rho C_{p} \right){}_{e\mathrm{ff}}$ is the effective volumetric heat capacity at constant pressure, the equivalent thermal conductivity $k_{e\mathrm{ff}}$ is the weighted equivalent of the averaging model of water and porous media. The $\theta_{s}$ is the porosity of the solar evaporator.

**S1.3 Solar steam generation test**

Solar radiation was simulated using a solar simulator (CEL-PE300L-3A, xenon lamp) with AM1.5G filter, the optical density of sunlight exposure was determined using an auxiliary detector (CEL-NP2000, optical power meter, China), and the optical density on the sample was adjusted by controlling the power and optical distance of the solar simulator. The spot size was controlled by the pore size to ensure that the spot size was equal to the sample size. Each sample was illuminated for 60 minutes and the mass change was measured in real-time using a computer-connected electronic balance (accuracy 0.1 mg), and the temperature was measured with an infrared camera. The real-time change in water evaporation quality recorded by the balance calculates the water evaporation rate under one-sun illumination (Table S2). In general, the evaporation area of a material (Ae) was equal to the light projection area (Ap). Therefore, the evaporation rate V can be calculated by the following equation:

$V=\frac{1}{Ae}\frac{dm}{dt}=\frac{M}{Ap}$ (S8)

where m is the mass of evaporated water and t is the lighting time. M is the mass of evaporated water, expressed as the slope of the mass loss curve obtained from a linear fit at steady state.

**S1.4 Anti-biofouling property**

The antimicrobial properties of the different samples were evaluated using Escherichia coli (E. coli, Gram negative) and Staphylococcus aureus (S. aureus, Gram positive), respectively. E. coli and S. aureus samples were removed from the -80°C refrigerator. An appropriate amount of the bacterial solution was added to 5 ml of broth medium, which was then placed in a thermostatic shaker and shaken at 220 revolutions per minute for 14 hours at 37 degrees Celsius. Next, the bacterial solution was diluted to 1x10^5^ per milliliter using phosphate buffered saline, 400 microliters of the bacterial solution was taken and mixed with aerogel, and the mixture was subsequently incubated in a thermostat at 37 degrees Celsius for 14 hours. Then, 30 microliters of the bacterial solution obtained from the incubation was evenly spread on agar medium and left overnight (14 hours) at 37 degrees Celsius. Finally, the colonies in each petri dish were counted to assess the antimicrobial effect of the samples. The antibacterial efficiency (R) was calculated using the following equation:

$R=\frac{Nc-Nm}{Nc}\times100\%$ (S9)

where *Nc* and *Nm* represent the number of colonies with the blank sample and PVA/SA、PVA/SA/MS , respectively.

**S2 Supporting Notes**

**Note S1 Crosslinker performance Test**

The macroscopic photographs of pure PVA aerogel after preparation are shown in Figure S1, and it can be clearly seen that the diameter and height of the pure PVA aerogel taken out of the lyophilizer have obviously shrunk in diameter and height, compared with the shape of the gel-like solid at the beginning of the completion of freeze-drying. One day after the removal, the original diameter of 2.4 cm of pure PVA aerogel was gradually reduced to 2.0 cm, 4 days after the removal, the diameter of pure PVA aerogel was reduced to 1.8 cm, and 7 days later, the diameter of pure PVA aerogel was only 1.6 cm. The height of pure PVA was shrunk at the same time, and the height of pure PVA aerogel with a height of 2 cm was reduced to 1.8 cm after 4 days, and 1.5 cm after 7 days. This may be due to the relatively poor mechanical properties and shrinkage of the pure PVA aerogel due to poor hydrogen bonding within the aerogel.

In order to solve the problem of shrinkage of PVA aerogel in air, silane coupling agent was added to PVA to increase the hydrogen bonding force between molecular chains in the aerogel. It can be seen that the shape of the PVA aerogel after the addition of silane coupling agent has been kept intact, and there is no change in the shape of the aerogel after taking it out of the lyophilizer, and the aerogel still maintains the original size after 7 days, which solves the shrinkage problem of the PVA aerogel.

**Note S2 Measurement of effective porosity of PSMS evaporator and cotton swab**

The dry PSMS evaporator and cotton swab were weighed using an electronic balance. After soaking in water for 10 minutes until completely wet, they were again weighed separately. Effective porosity of the evaporator and cotton swab can be calculated using equation S8:

$Q\left( \% \right)=\frac{(m_{2}-m_{1})/\rho_{2}}{m_{1}/\rho_{1} +(m_{2}-m_{1})/\rho_{2}}$ (S10)

where m_1_ and m_2_ are the evaporated weights (G) of the samples before and after wetting, respectively, and ρ_1_ and ρ_2_ are the densities of the samples (PSMS: 21.5 kg m^-3^ Cotton swabs: 167.6 kg m^-3^) and water (1.0×10^3^ kg m^-3^), respectively. To reduce the experimental error, evaporation was measured three times to take the average value.

**Note S3 Calculation of permeability, volumetric flow rate and darcy velocity**

Based on the SEM images for 2D modeling, the geometry and boundary conditions of the fully resolved microscale model solved through the peristaltic flow module using the peristaltic flow interface were used to calculate the permeability, volumetric flow rate, and Darcy's velocity of the PSMS-1 aerogel and cotton rods, respectively.

**Note S4** **Energy gain in the cold region of evaporating surface**

Energy gain (𝑄_𝑔𝑎𝑖𝑛_) from environment at cold surface can be calculated by following equations∶

$Q_{gain}=-A_{c}h\left( T_{c}-T_{a} \right)-\varepsilon\sigma A_{c}\left( T_{c}^{4}-T_{a}^{4} \right)$ (S11)

Where,

𝐴_𝑐_ = Area of the cold region of the evaporating surface

𝑇_𝑐_ = Average temperature of the cold region of the evaporating surface

𝑇_𝑎_ = Ambient temperature

ℎ = Convective heat transfer coefficient of air in natural convection = 8 W m^-2^K^-1^

𝜎 = Stefan-Boltzmann constant = 5.67 $\times$ 10-8 W m^-2^K^-4^

ε = Emissivity of the evaporator’s surface = 0.85

For PSMS-1 evaporator under 1 sun illumination, A_c_ = 0.00157 m^2^, T_a_ ≈ 299.85 K, T_c_ ≈ 289.35 K. From the above-mentioned equations the Q_gain_ ≈ 0.213 W

**Note S5** **Solar-to-vapor energy conversion efficiency calculation**

The solar-to-vapor energy conversion efficiency can be calculated by

$\eta=\frac{V_{n}\left( H_{lv}+Q \right)}{E_{i}}$ (S12)

$\text{H}_{l\text{v}}\text{=1}\text{.}\text{91846×}\text{10}^{\text{6}}\text{×}\left( \frac{\text{T}_{\text{a}}}{\text{T}_{\text{a}}\text{-33}\text{.}\text{91}} \right)$ (S13)

$\text{Q=c×}\left( T_{a}-T_{i} \right)$ (S14)

Where,

v_n_= net evaporation rate (kg m^-2^ h^-1^)

v_n_= v_l_$-$v_d_

In these equations

V_d_= Dark evaporation rate (kg m^-2^ h^-1^)

𝐸_𝑖_= Energy input of the incident light (kJ m^-2^ h^-1^)

H_lv_= Latent heat required for vaporization of water (J kg^-1^)

T_a_= Average temperature of the solar evaporation surface (K)

Q= Required heat for increasing the temperature of water

c= Specific heat of water (4.2 J g^-1^ K^-1^)

T_i_= Initial average temperature of top surface (K)

For PSMS-1 evaporator under 1 sun illumination, T_a_ ≈ 299.85 K, T_i_ ≈ 286.35 K, v_l_ ≈ 3.6 kg m^-2^ h^-1^, v_d_ ≈ 0.87 kg m^-2^ h^-1^. From the above-mentioned equations the solar-to-vapor energy conversion efficiency becomes ≈ 189%.

**S3 Supplementary Figures**

**
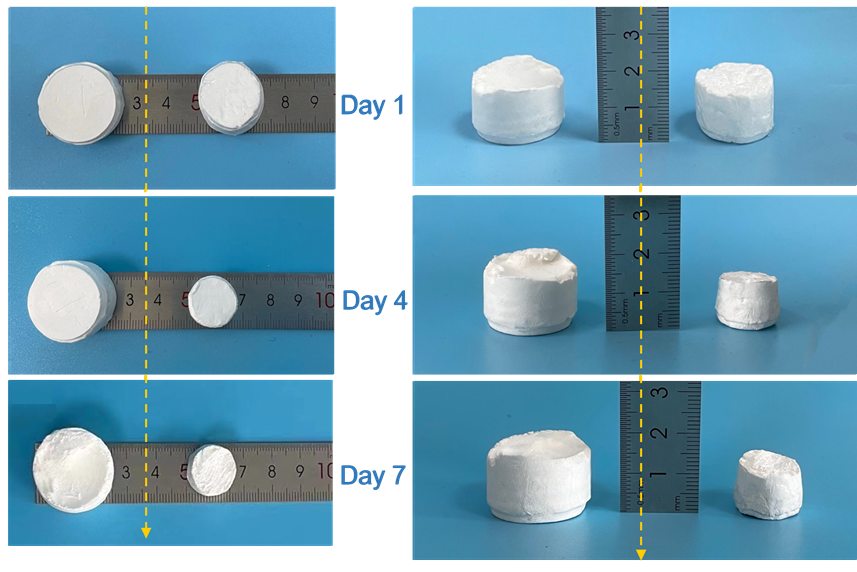
**

**Fig. S1** PVA aerogel with silane coupling agent (left) versus pure PVA aerogel (right)


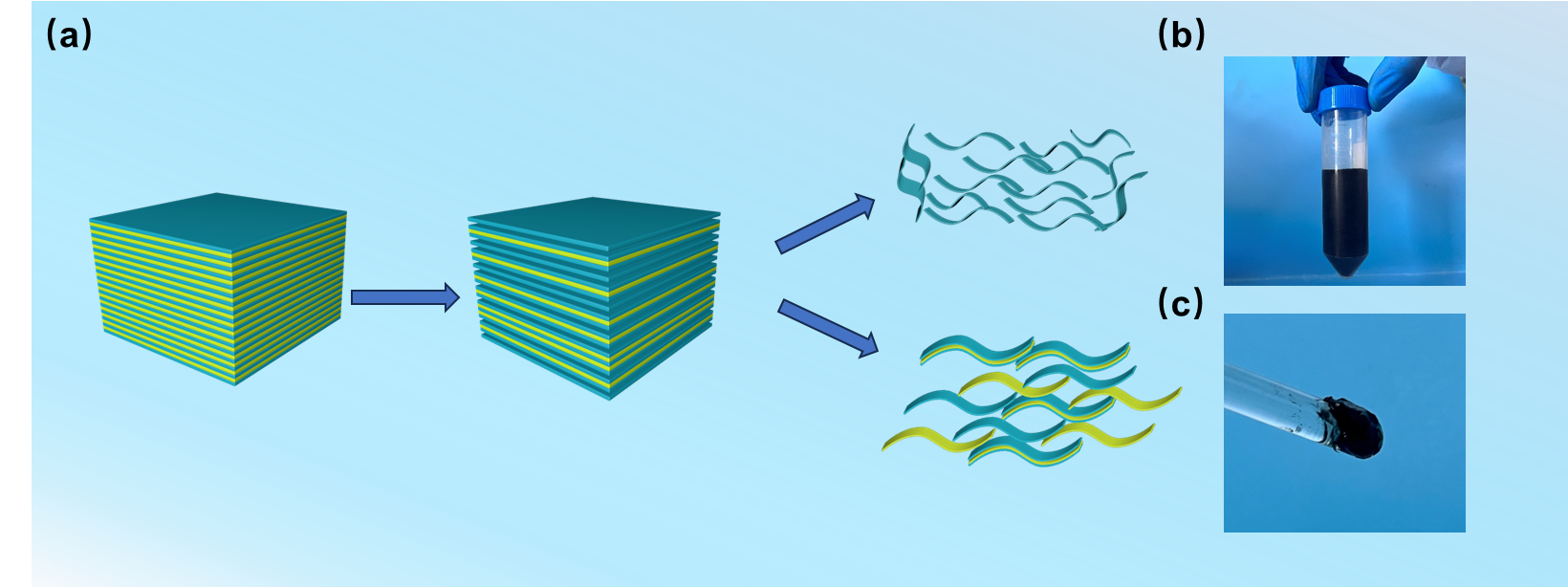


**Fig. S2** **a** Schematic of the fabrication of ultrathin Ti_3_C_2_T_x_ MXene nanosheets and MXene sedimengt. **b, c** Digital image of an aqueous solution of MXene nanosheets (above) and MXene sediments (under)


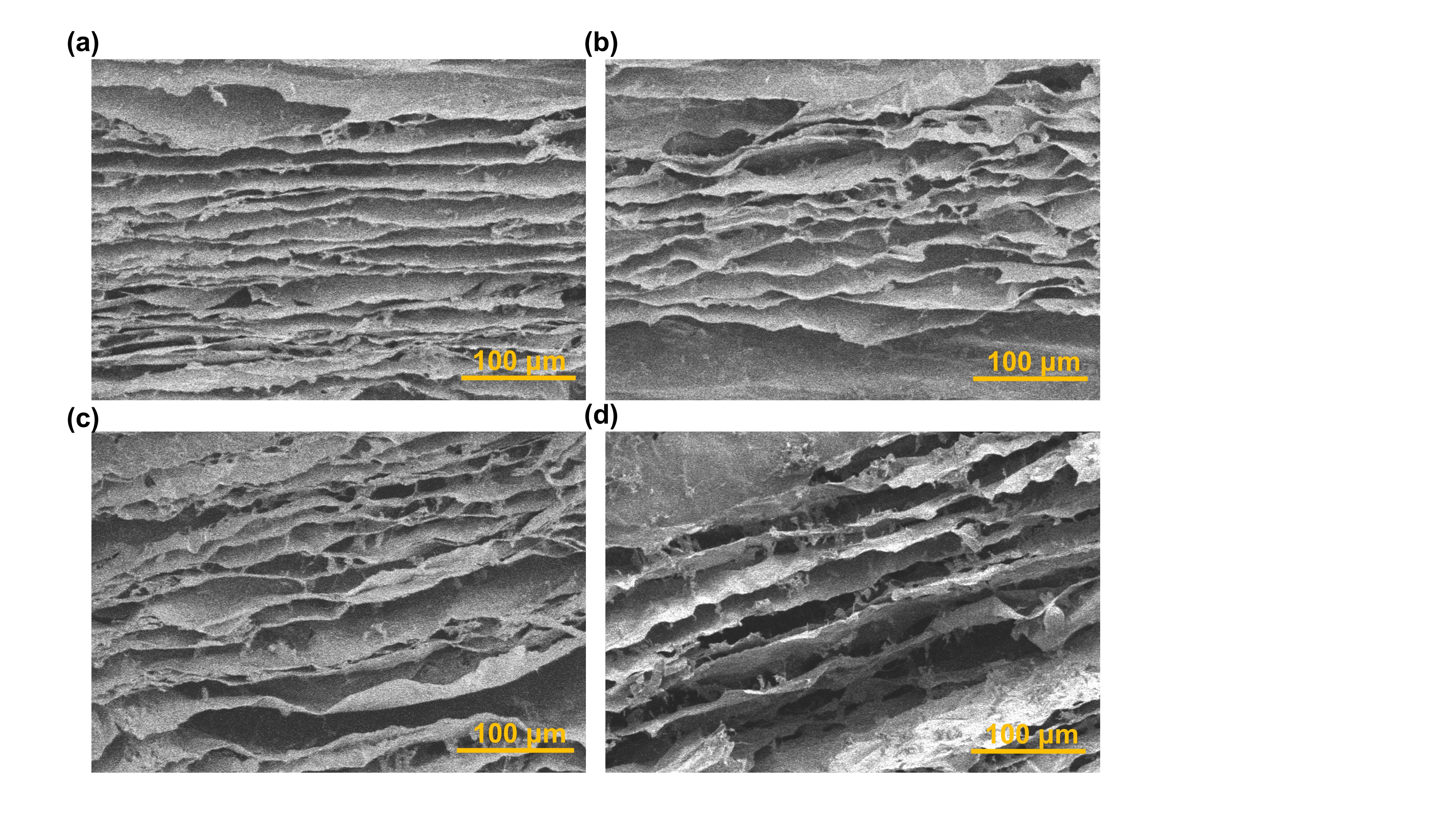


**Fig. S3** PSMS aerogel in the longitudinal direction was observed under SEM


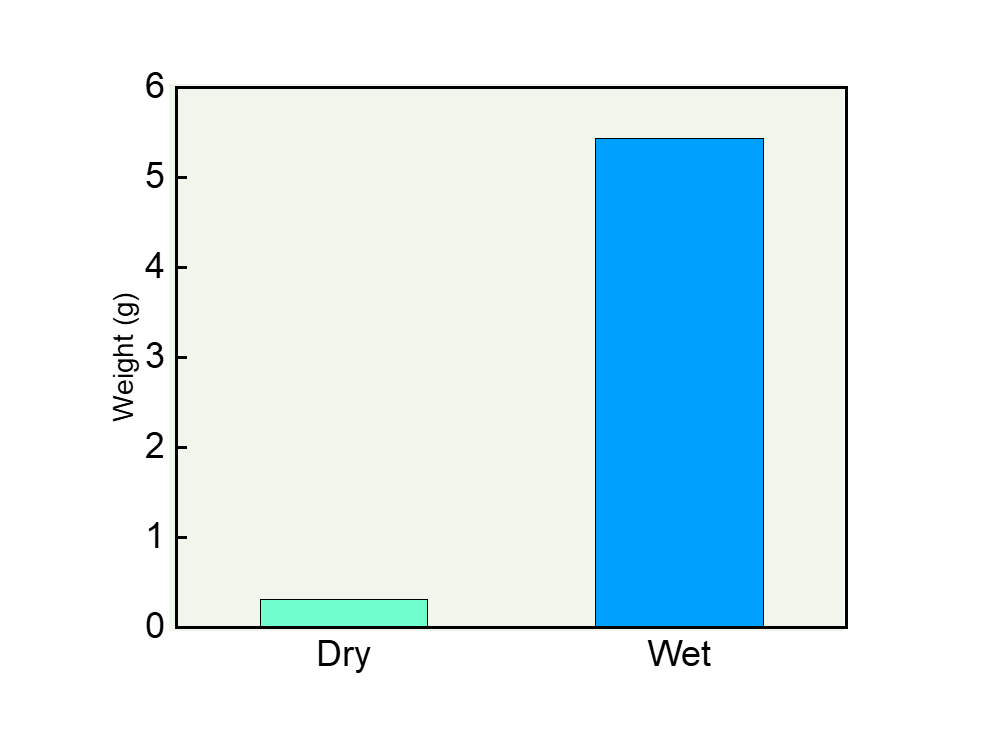


**Fig. S4** Density of PSMS aerogel before water absorption and after full saturation


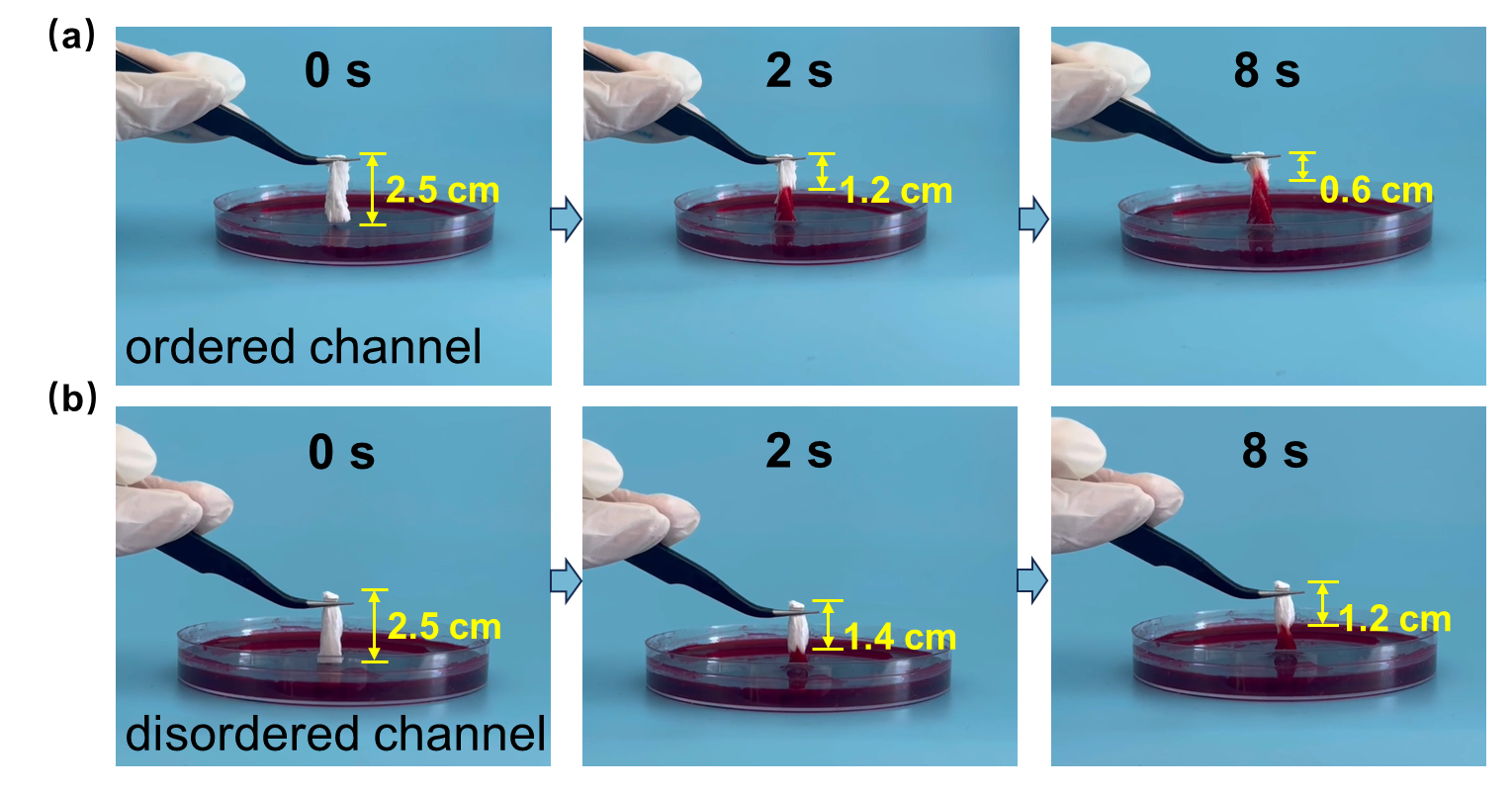


**Fig. S5 a, b** Water transport image and curves of the fluidic height over time of PSMS aerogel strips with/without the directed channel


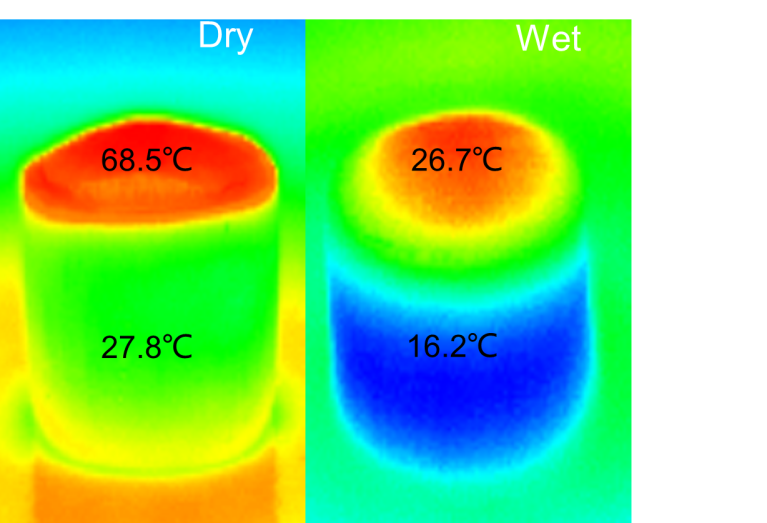


**Fig. S6** PSMS-1 infrared image of the evaporator in wet and dry state


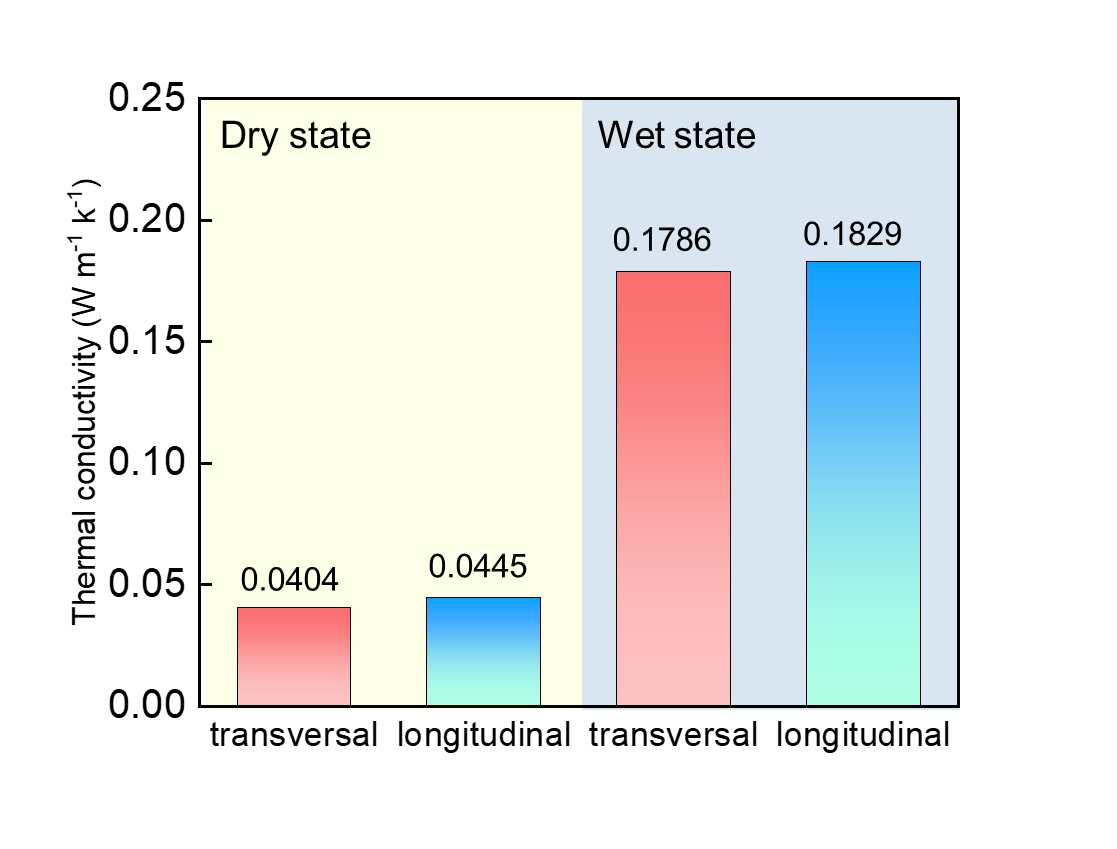


**Fig. S7** Longitudinal and transverse thermal conductivities of PSMS-1 evaporator in dry and wet states

**
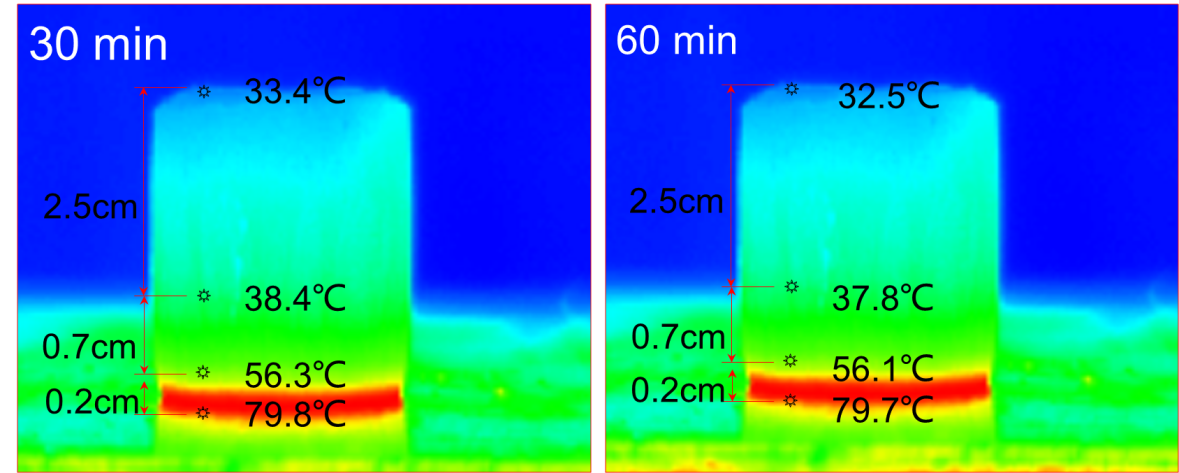
**

**Fig. S8** Infrared images of PSMS-1 evaporator in dry state at 30 and 60 minutes on the hot bench


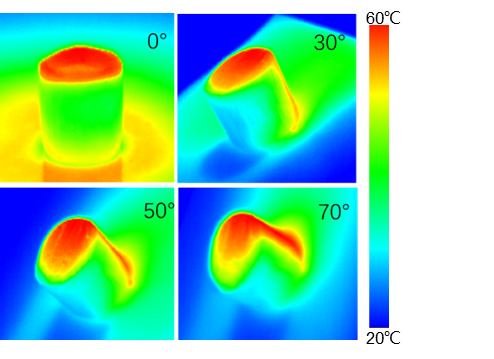


**Fig. S9** Infrared images of the PSMS-1 evaporator under sunlight at different angles of incidence


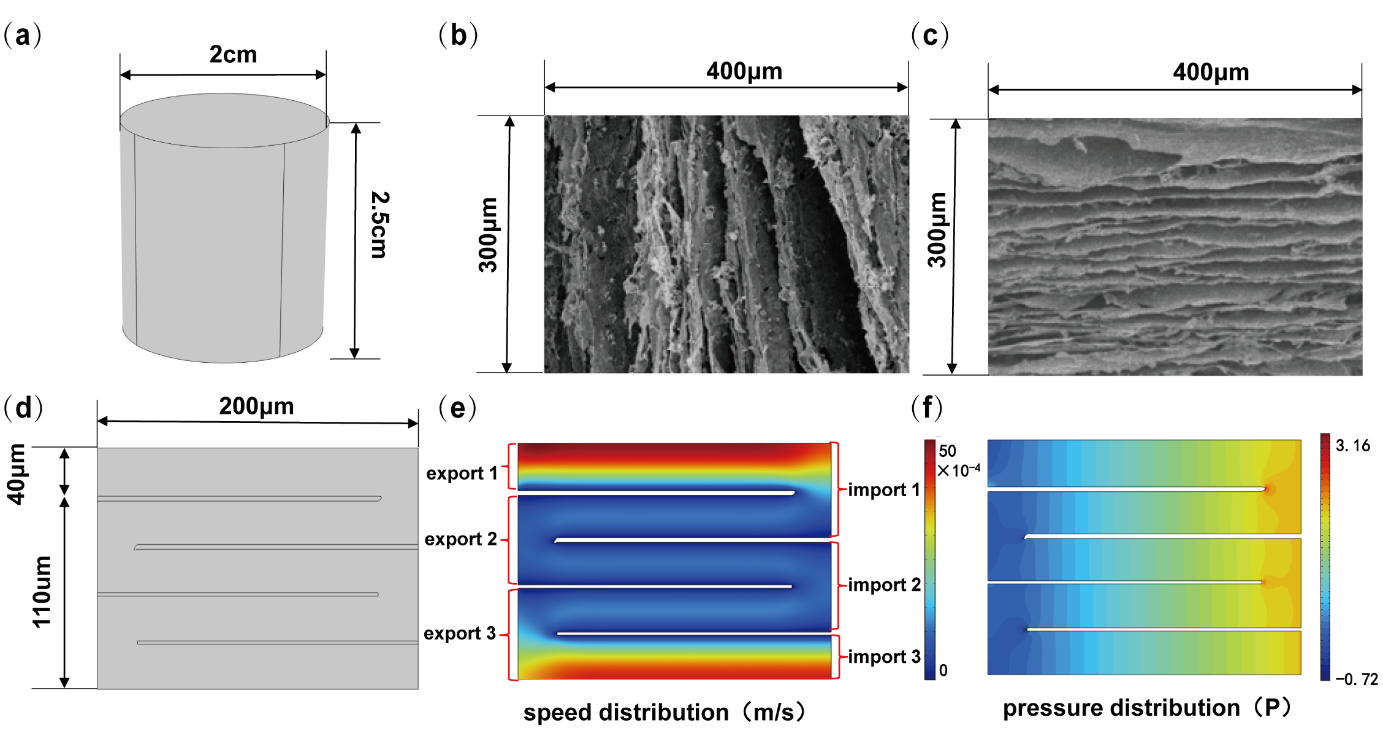


**Fig. S10** **a** Modeling of PMSM-1 aerogel. **b, c** SEM images of PSMS-1 aerogel. **d** Modeling of channels inside the evaporator. **e** Velocity discretization inside the evaporator. **f** Pressure distribution inside the evaporator


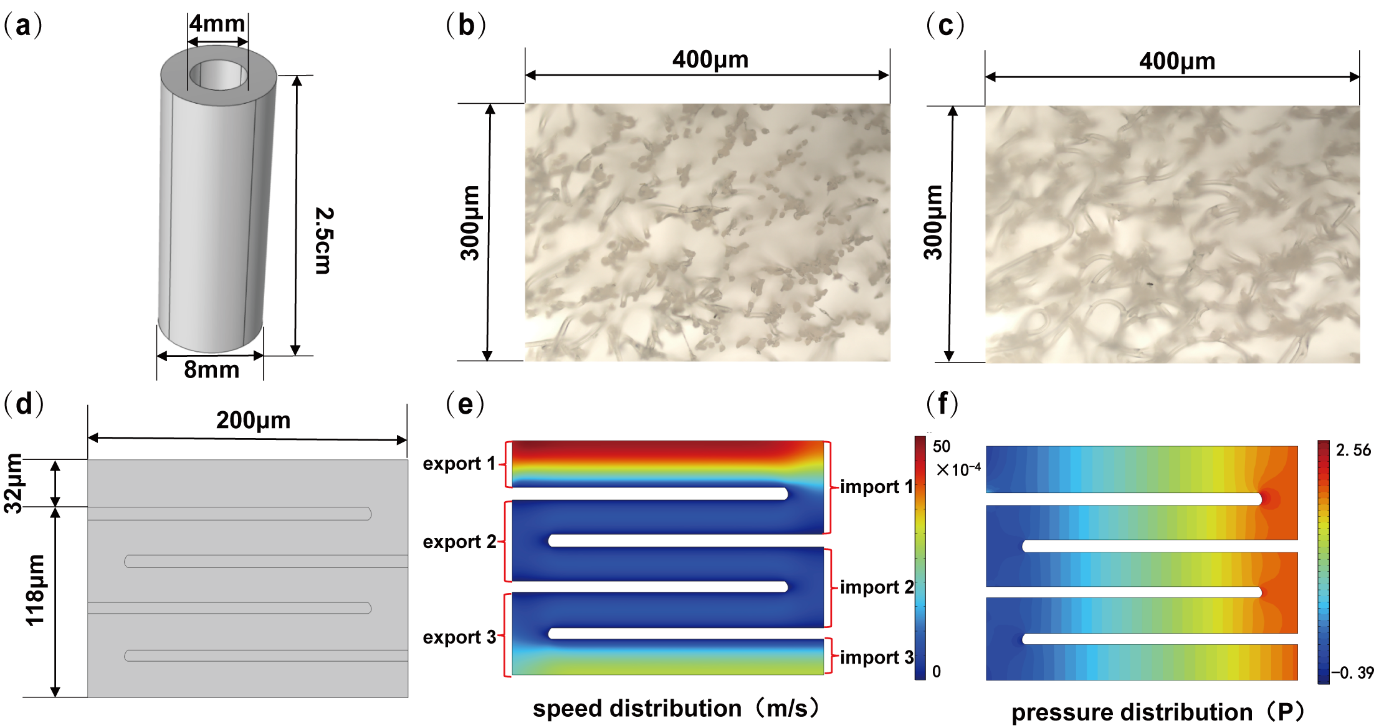


**Fig. S11** **a** Modeling of water-conveying swabs. **b, c** SEM images of swabs. **d** Internal channel model of swabs. **e** Velocity discretization inside swabs. **f** Pressure distribution inside swabs


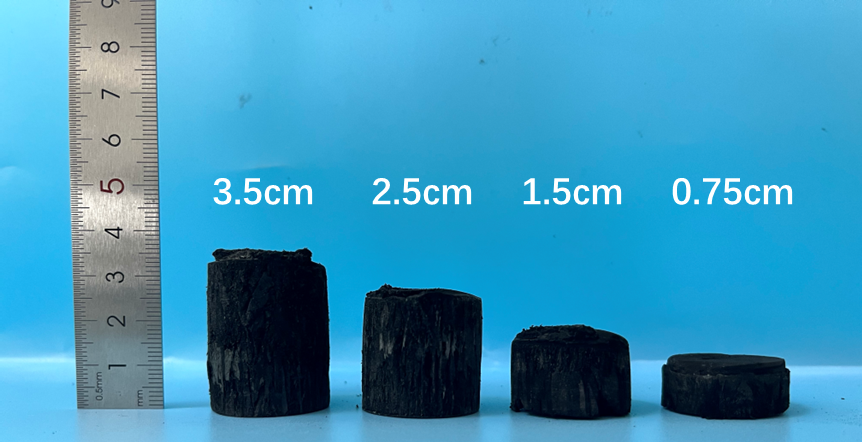


**Fig. S12** PSMS evaporators at different heights (3.5, 2.5, 1.5, 0.75cm)

**
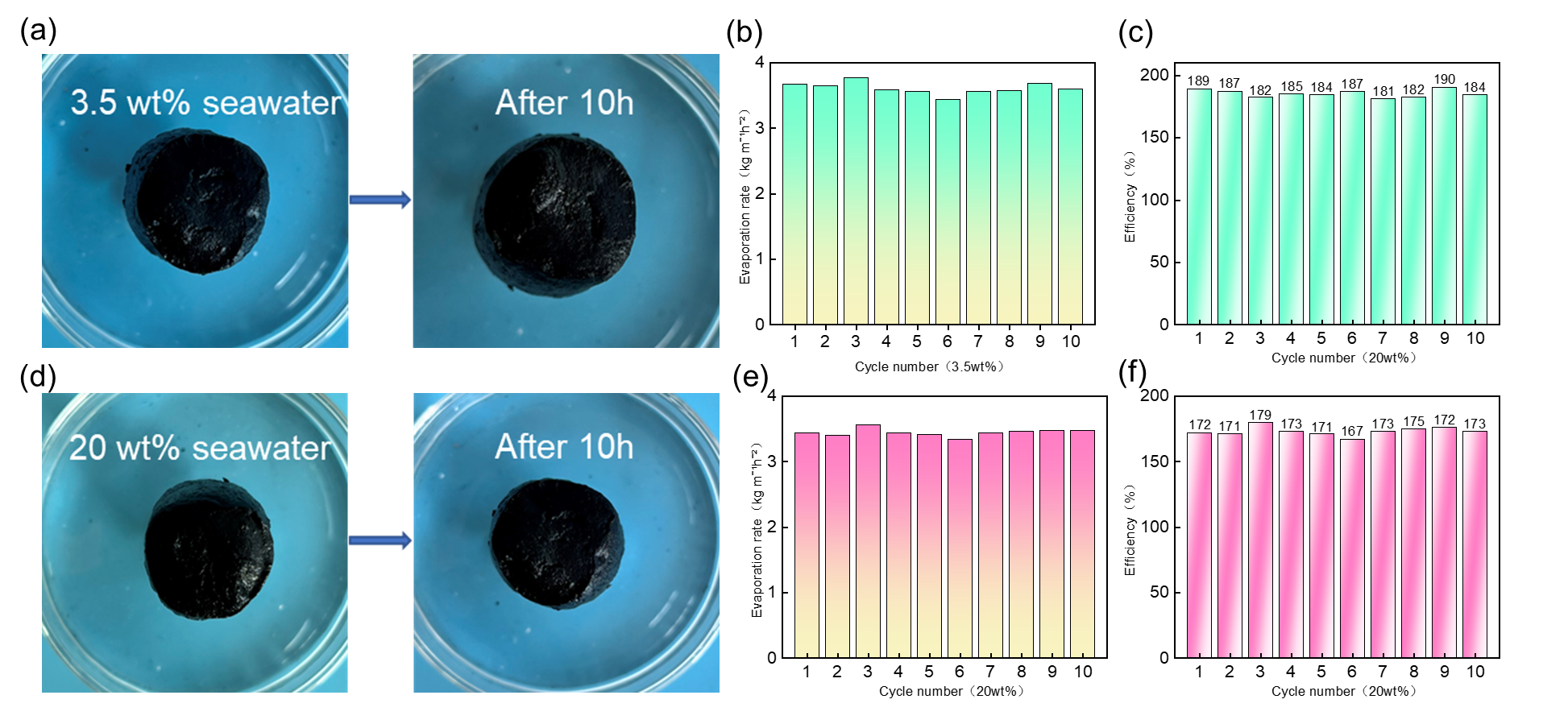
**

**Fig. S13** **a** Evaporator (1 sunlight) evaporated in 3.5 wt% brine for 10 h surface contrast. **b, c** Evaporation rates and efficiencies for 10 runs of 3.5 wt% brine. **d** Evaporator (1 sunlight) evaporated in 20 wt% brine for 10 h surface contrast. **e, f** Evaporation rates and solar-to-vapor energy conversion efficiencies for 10 runs of 20 wt% brine


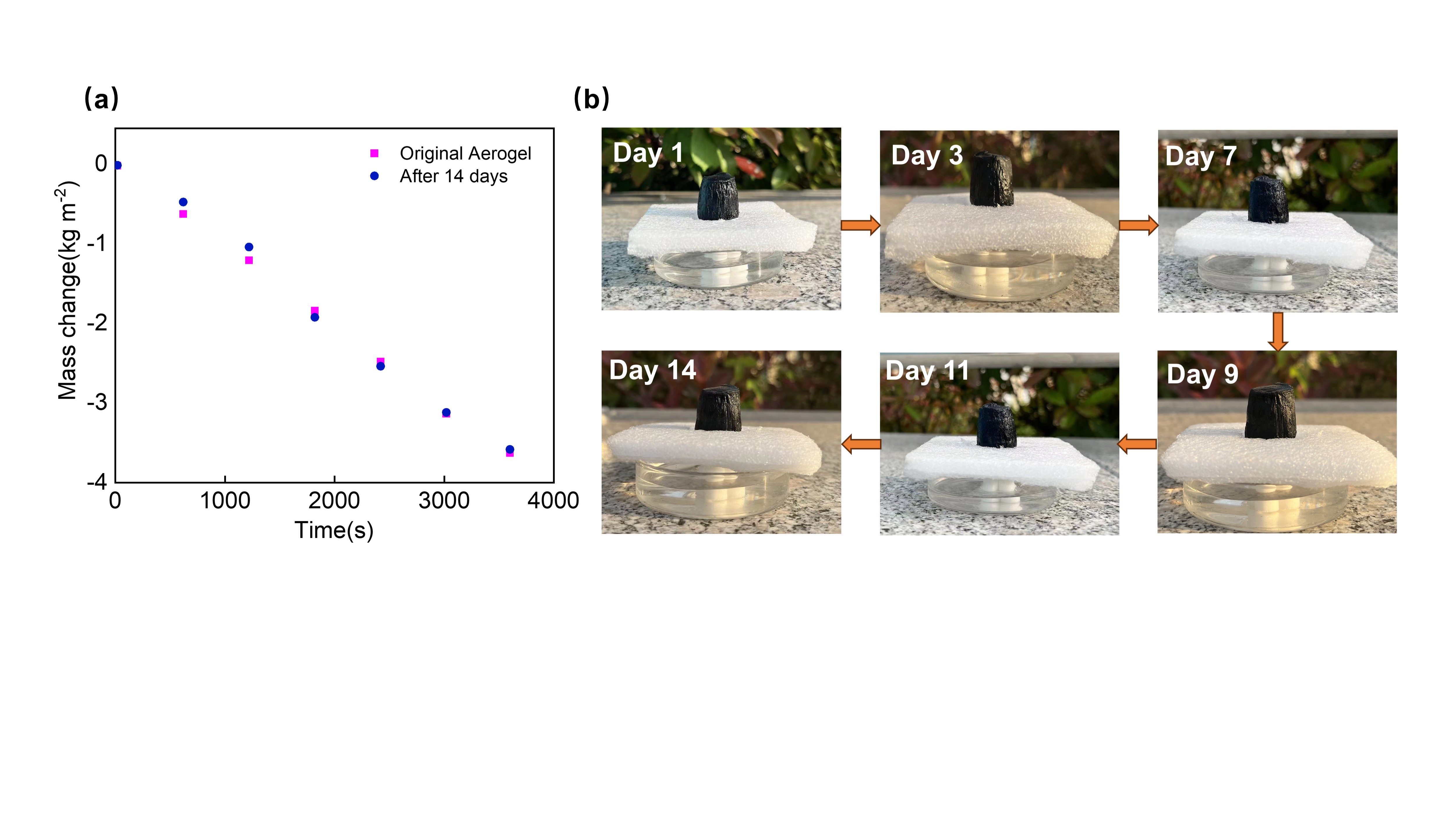


**Fig. S14** **a** Comparison of evaporation rates before and after 14 days. **b** The PSMS evaporator with a height of 2.5 cm evaporated natural seawater under natural light without salt crystals on the surface


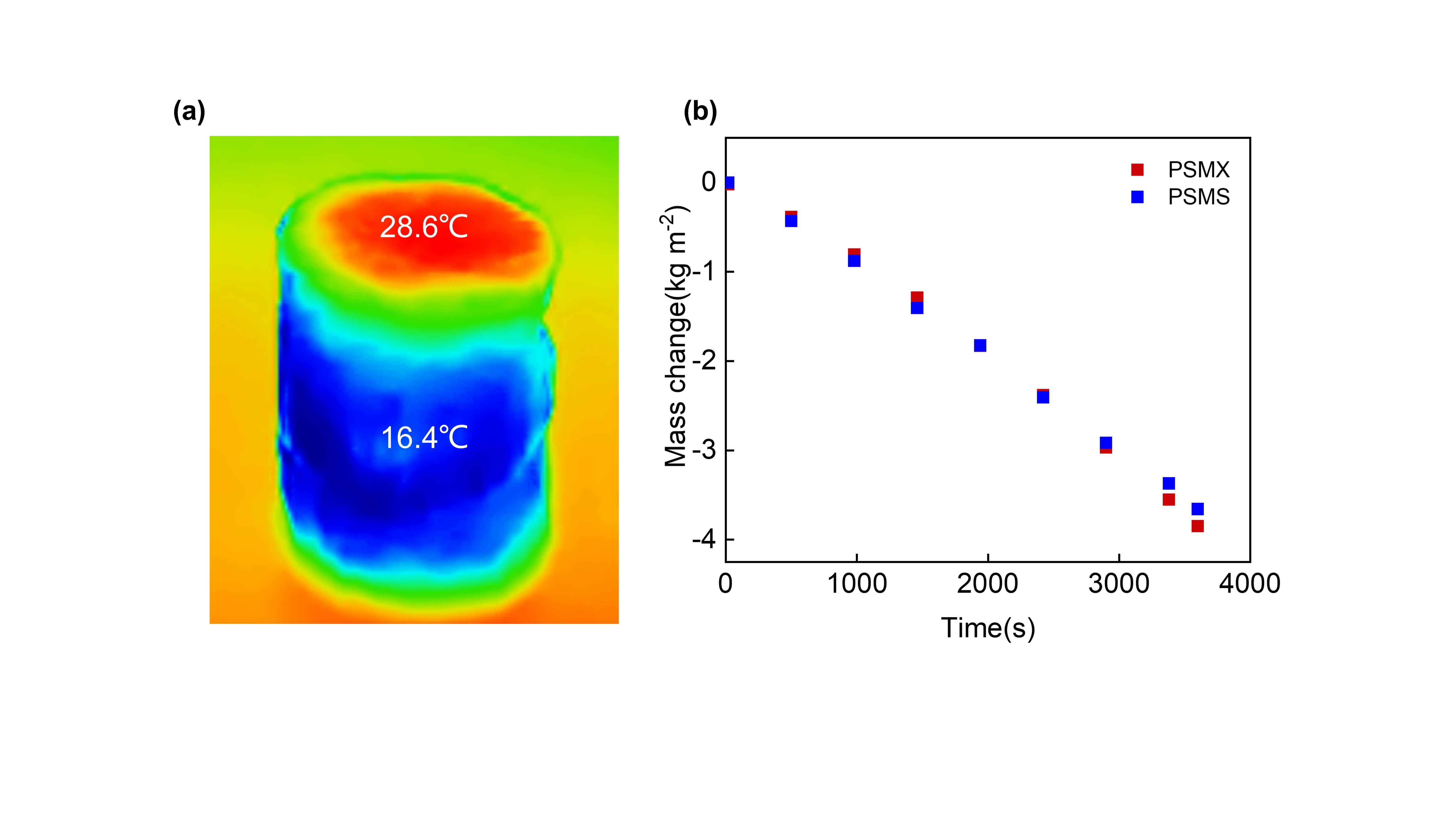


**Fig. S15 a** Infrared image of PSMX aerogel under 1 sunlight irradiation. **b** Evaporation rates of PSMS and PSMX under 1 sunlight irradiation


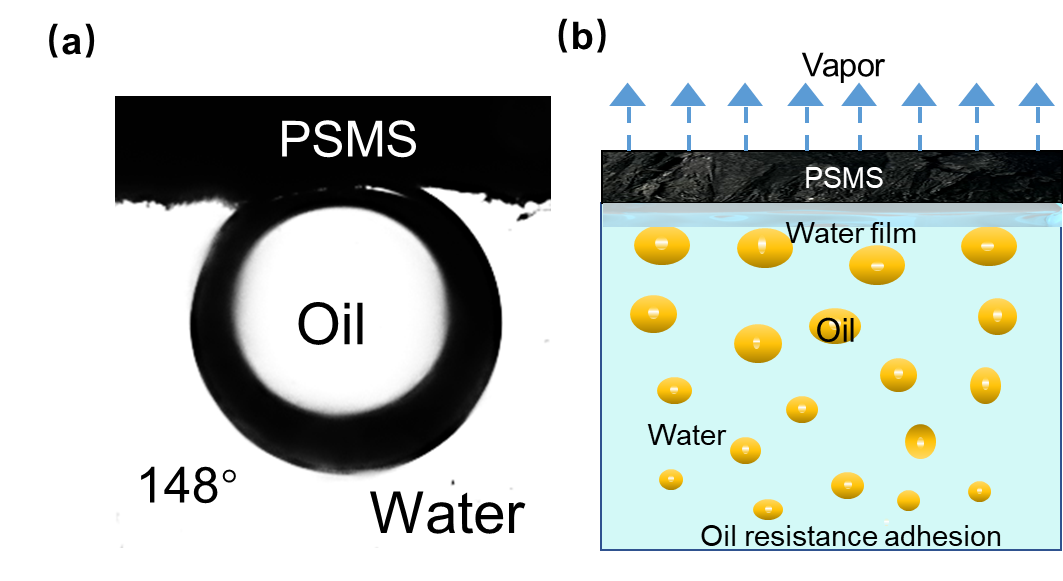


**Fig. S16** **a** Underwater oil contact angle of PSMS evaporator. **b** Schematic representation of the excellent underwater oil resistance of the PSMS evaporator


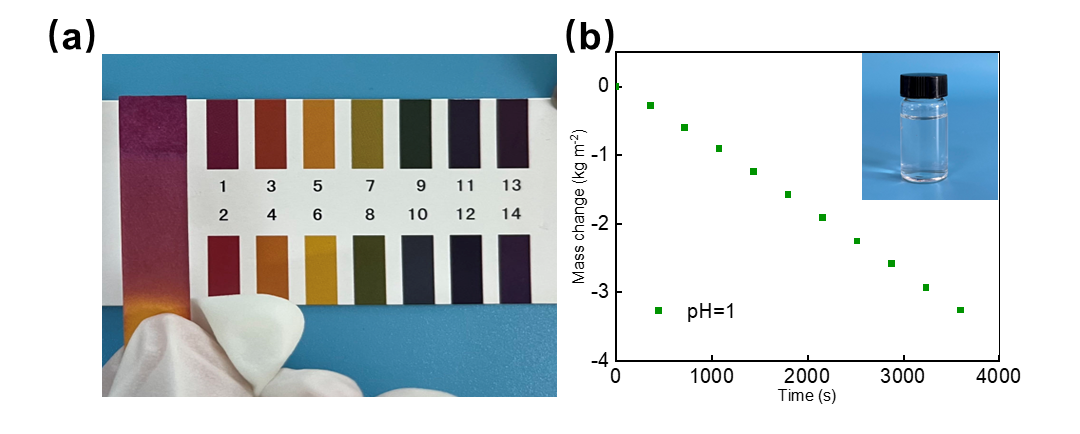


**Fig. S17** **a, b** Water weight loss of a PSMS-1 evaporator in HCl solution (PH=1) under simulated sunlight


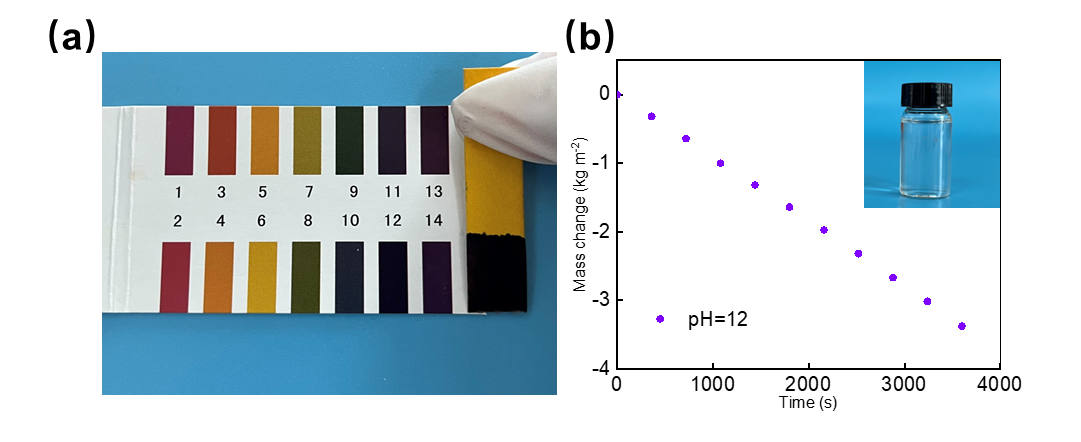


**Fig. S18** **a, b** Water weight loss of a PSMS-1 evaporator in NaOH solution (PH=12) under simulated sunlight


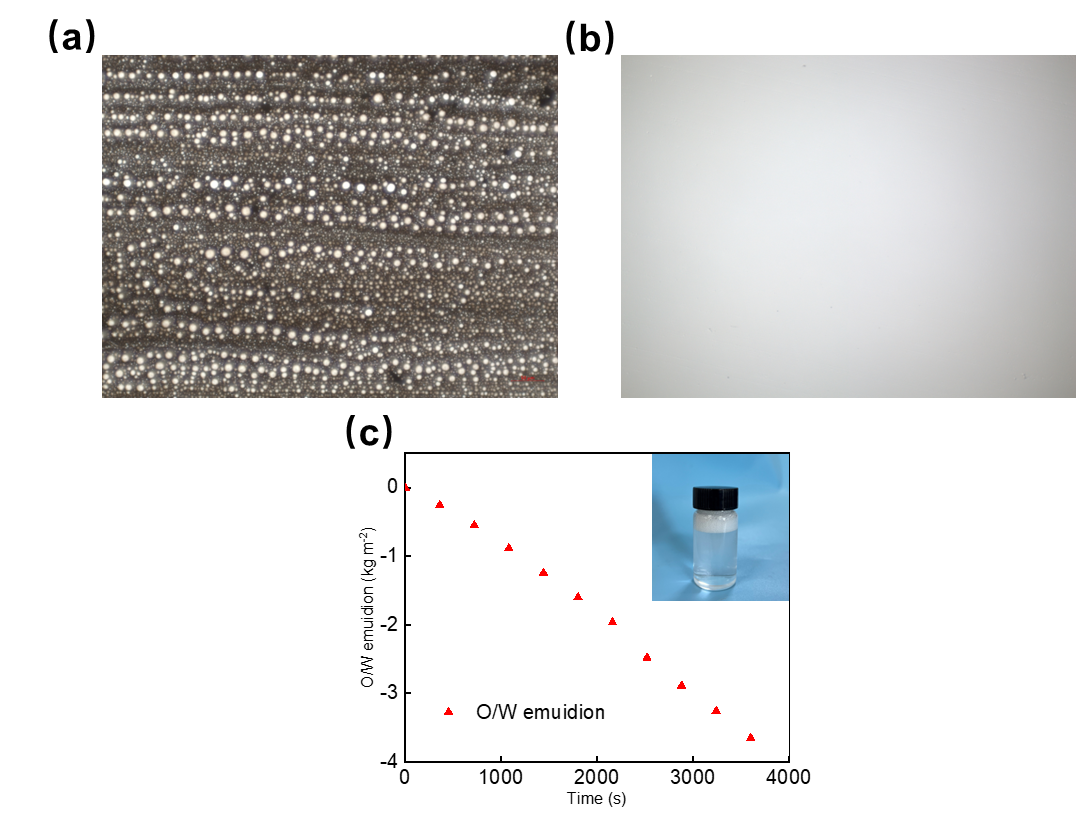


**Fig. S19** **a** Optical microscope image of emulsified oil/water mixture before evaporator-driven separation. **b** Photograph of emulsified oil/water mixture after evaporator-driven separation. **c** Loss of weight of emulsified oil/water mixture in PSMS-1 evaporator under simulated sunlight


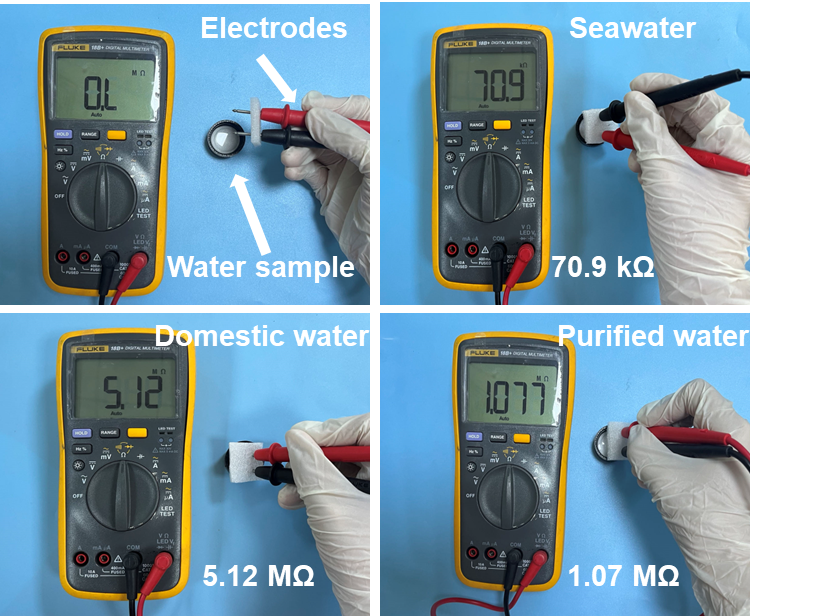


**Fig. S20** The resistance of different water measured by a multimeter with constant electrode spacing

**S4 Supplementary Tables**

**Table S1** Materials.

| Reagent name | Specification |  | Manufacturer |
| --- | --- | --- | --- |
| Ti_3_AlC_2_ | 400 mesh |  | Jilin Province 11 Technology Co., Ltd |
| HCl | 37% |  | Shanghai Aladdin Biochemical Technology Co., Ltd |
| LiF | Analyze pure |  | Shanghai Aladdin Biochemical Technology Co., Ltd |
| Cotton swabs | Diameter 1-8 mm |  | Dongguan Zhengfeng Foam Products Co., Ltd |

**Table S2** PSMS aerogels with different PVA and SA concentrations

| Sample | PVA | SA | MXene sediments |
| --- | --- | --- | --- |
| PSMS-1 | 5 mL 1wt%PVA | 15 mL 1wt%SA | 3.6 g |
| PSMS-2 | 5 mL 2wt%PVA | 15 mL 2wt%SA | 3.6 g |
| PSMS-3 | 5 mL 3wt%PVA | 15 mL 3wt%SA | 3.6 g |
| PSMS-4 | 5 mL 4wt%PVA | 15 mL 4wt%SA | 3.6 g |
| PSMS-5 | 5 mL 1wt%PVA | 15 mL 1wt%SA | 2.6 g |
| PSMS-6 | 5 mL 1wt%PVA | 15 mL 1wt%SA | 4.6 g |

**Table S3** Experimental instruments

| Instrument name | Model | Manufacturer |
| --- | --- | --- |
| Xenon light source | CEL-PE300L-3A | Beijing Zhongjiao Jinyuan Technology Co., Ltd |
| Optical power density meter | CEL-NP2000 | Beijing Zhongjiao Jinyuan Technology Co., Ltd |
| Freeze dryer | FDU-2110 | Pikakikal in Tokyo, Japan |
| UV/VIS near-infrared spectrophotometer | UV-3600 | Shimadzu Corporation of Japan |
| Scanning electron microscope | ZEISSEVO18 | Carl Zeiss, Germany |
| Contact angle tester | OCA 15EC | German company Dataphysics |
| X-ray photoelectron spectroscopy | Escalab 250 Xi | American company Thermo Scientific |
| Inductively coupled plasma-optical emission spectrometry | Avio 200 | Perkin Elmer |
| Thermal conductivity meter | TC3000E | Xian Xiaxi Technology Co., Ltd |

**Table S4** PSMS-1 evaporator model parameters

| Causality | | numerical value | work unit |
| --- | --- | --- | --- |
| Densities | | 21.5 | kg m^-3^ |
| Constant pressure heat Capacity | | 1008.74 | J kg^-1^ K^-1^ |
| Porosity | | 0.94 |  |
| Permeability | 1.5$\times$10^-10^ | m^2^ |  |
| Thermal conductivity | | 0.1823 | W m^-1^ K^-1^ |
| Volumetric flow | | 2.3$\times$10^-7^ | m^3^/s |
| Darcy Speed | | 1.56$\times$10^-3^ | m/s |

**Table S5** Cotton swab model parameters

| Causality | numerical value | work unit |
| --- | --- | --- |
| Densities | 167.6 | kg m^-3^ |
| Constant pressure heat Capacity | 973.9 | J kg^-1^ K^-1^ |
| Porosity | 0.82 |  |
| Permeability | 1.14$\times$10^-10^ | m^2^ |
| Thermal conductivity | 0.4478 | W m^-1^ K^-1^ |
| Volumetric flow | 1.718$\times$10^-7^ | m^3^/s |
| Darcy Speed | 1.14$\times$10^-3^ | m/s |

**Table S6** Comparison of 3D aerogel evaporators

| Compare samples | Material | Preparation method | Evaporation rate  (kg m^-2^ h^-1^) |
| --- | --- | --- | --- |
| Ref.1 | HPAN/PVA/CNTs | freeze drying | 2.33 |
| Ref.2 | CNF/MXene | freeze drying | 2.287 |
| Ref.3 | GO/MXene | freeze drying | 1.27 |
| Ref.4 | MXene/CTS | freeze drying | 1.27 |
| Ref.5 | PI/MXene | 3D-printed | 2.17 |
| Ref.6 | PVA/MXene | freeze drying | 2.71 |
| Ref.7 | VA-MXene | freeze drying | 1.46 |
| Ref.8 | PI/MXene/PEG | freeze drying | 1.24 |
| Ref.9 | SM-MXene/PVA | freeze drying | 1.8 |
| Ref.10 | MXene/PCM | freeze drying | 1.77 |
| This work | MS/SA/PVA | freeze drying | 3.6 |
